# Coupling PROSPECT with Prior Estimation of Leaf Structure to Improve the Retrieval of Leaf Nitrogen Content in *Ginkgo* from Bidirectional Reflectance Factor Spectra

**DOI:** 10.34133/plantphenomics.0282

**Published:** 2024-12-13

**Authors:** Kai Zhou, Saiting Qiu, Fuliang Cao, Guibin Wang, Lin Cao

**Affiliations:** Co-Innovation Center for Sustainable Forestry in Southern China, Nanjing Forestry University, Nanjing 210037, China.

## Abstract

Leaf nitrogen content (LNC) is a crucial indicator for assessing the nitrogen status of forest trees. The LNC retrieval can be achieved with the inversion of the PROSPECT-PRO model. However, the LNC retrieval from the commonly used leaf bidirectional reflectance factor (BRF) spectra remains challenging arising from the confounding effects of mesophyll structure, specular reflection, and other chemicals such as water. To address this issue, this study proposed an advanced BRF spectra-based approach, by alleviating the specular reflection effects and enhancing the leaf nitrogen absorption signals from *Ginkgo* trees and saplings, using 3 modified ratio indices (i.e., mPrior_800, mPrior_1131, and mPrior_1365) for the prior estimation of the N_struct_ structure parameter, combined with different inversion methods (STANDARD, sPROCOSINE, PROSDM, and PROCWT). The results demonstrated that the prior N_struct_ estimation strategy using modified ratio indices outperformed standard ratio indices or nonperforming prior N_struct_ estimation, especially for mPrior_1131 and mPrior_1365 yielding reliable performance for most constituents. With the use of the optimal approaches (i.e., PROCWT_S3 combined with mPrior_1131 or mPrior_1365), our results also revealed that the optimal estimation of LNC_area_ (normalized root mean square error [NRMSE] = 12.94% to 14.49%) and LNC_mass_ (NRMSE = 10.11% to 10.75%) can be further achieved, with the selected optimal wavebands concentrated in 5 common main domains of 1440 to 1539 nm, 1580 to 1639 nm, 1900 to 1999 nm, 2020 to 2099 nm, and 2120 to 2179 nm. These findings highlight marked potentials of the novel BRF spectra-based approach to improve the estimation of LNC and enhance the understanding of the impact of N_struct_ prior estimation on the LNC retrieval in leaves of *Ginkgo* trees and saplings.

## Introduction

*Ginkgo* (*Ginkgo biloba* L.) is an economically valuable tree species native to China, with its leaves rich in flavonoids, terpenoids, and other compounds that offer therapeutic and health benefits, particularly in the prevention of cardiovascular and cerebrovascular illnesses [1]. Nitrogen is a vital component of the vitamin, amino acid, and energy systems within plants [2]. It boosts the protein content in plants, facilitates chlorophyll synthesis, and enhances the process of photosynthesis. Nitrogen is essential for the growth and development of forest trees and plays a crucial role in *Ginkgo* growth [3]. In early spring (March), nitrogen is applied as a basal fertilizer to support bud development. During the rapid growth stage (late May to July), nitrogen fertilizer serves as top-dressing to promote vigorous shoot branching and leaf expansion. From late July to September, additional nitrogen is applied as top dressing to enhance leaf thickness, increasing the photosynthesis and the accumulation of medicinal components in the later growth phase (i.e., plant maturity stage) [4,5]. However, excessive or insufficient nitrogen fertilizer application can negatively impact tree health, highlighting the importance of optimal nitrogen levels. Due to limited understanding of nitrogen demands by *Ginkgo* growers, production practices for *Ginkgo* often rely on visual diagnosis and empirical measurement, potentially leading to overfertilization, environmental pollution, and even a reduction in biomass. Timely and accurate nitrogen nutritional assessment is therefore essential for promoting the healthy growth of *Ginkgo* and minimizing these risks. While standard methods for nitrogen assessment involve sampling *Ginkgo* leaf tissues at key growth stages and analyzing nitrogen content through laboratory protocols [4,5], visual diagnosis typically identifies nutrient imbalances only after they have visibly impacted the plant. In contrast, remote sensing techniques can enhance nitrogen assessment by detecting subtle physiological changes prior to the appearance of visible symptoms [6]. This capacity allows for earlier intervention and more comprehensive evaluations of nitrogen status. By employing remote sensing to estimate nitrogen content from leaf samples at critical growth stages under varying nitrogen fertilizer levels, timely nondestructive nitrogen assessments for *Ginkgo* trees and saplings can be potentially achieved.

Leaf nitrogen content (LNC) is widely used as an evaluation indicator for assessing nitrogen nutrition in forest trees. The traditional laboratory chemical analysis method (such as micro-Kjeldahl method) for the LNC measurement is accurate but complex, involving destructive sampling requirements, intricate steps like acid digestion and distillation, and a delayed result delivery, all of which require careful handling and technical expertise [6]. Previous studies have indicated that spectroscopic techniques can estimate LNC quickly and nondestructively [7], primarily through statistical modeling methods based on a single-leaf reflectance spectral analysis. However, regression models are influenced by various factors, such as sample size [8], species [9], season (or leaf age) [10], and illumination–observation geometry [11]. The latter significantly affects the specular reflectance spectra from the leaf surface, particularly in the backward scattering direction. These above factors can reduce the generalizability of regression models, whereas mechanistic models provide a more generalizable approach [2]. Furthermore, hybrid approaches that combine radiative transfer models (RTMs) with data-driven methods have been employed to improve predictions of leaf biochemical traits (e.g., LNC), by training machine learning regression models on RTM-derived biochemical parameters [9,10,12]. While spectral metrics in the visible and red-edge bands were highly related to chlorophyll features and have been used in estimating LNC in numerous studies, the relationship between chlorophyll and nitrogen is nonlinear. This phenomenon arises from the metastability of nitrogen across various plant organs and its dependence on reproductive periods and ecological environments [13]. As a result, changes in chlorophyll content do not occur concurrently with nitrogen variation, leading to delayed fluctuations in chlorophyll levels and an inconsistent relationship between chlorophyll content and nitrogen levels. Consequently, relying on chlorophyll-related spectral metrics to determine nitrogen status may result in delayed effects.

The chlorophyll in leaves contains less than 2% of the total nitrogen present, while proteins are the most important nitrogen-containing biochemical parameters. For instance, the ribulose-1,5-bisphosphate carboxylase/oxygenase (enzyme RuBisCO), which is found in green leaves, contains 30% to 50% of the nitrogen present [14,15]. While chlorophyll content is generally correlated with RuBisCO levels, it is important to recognize that chlorophyll is not the major reservoir of nitrogen in leaves. This correlation is not always consistent, such as the delayed response of chlorophyll to nitrogen status can complicate the accurate estimation of nitrogen content based solely on chlorophyll metrics. As highlighted by Peanusaha et al. [10], although chlorophyll or leaf greenness can serve as a proxy for nitrogen content, it may not reliably reflect actual nitrogen levels under varying conditions. In particular, the enzyme plays a crucial role in the rate of carbon assimilation in photosynthesis and serves as an important source for nitrogen remobilization and translocation within the plant. Yeoh and Wee [16] conducted a study on 90 plant types and found a reliable conversion factor of 4.43 between LNC and protein content. Wang et al. [17] used airborne hyperspectral imagery to estimate nitrogen concentration in forest leaves and discovered that the short-wave infrared band, which contains the protein absorption feature, was more sensitive to LNC and can significantly improve the estimation accuracy. Therefore, spectral techniques offer a reliable and nondestructive method for assessing nitrogen status, based on the estimation of LNC converted from protein content [18].

In the past 2 decades, mechanistic models have been widely used to estimate vegetation biochemical parameters. These models are based on the interaction between light and various components of vegetation. They can simulate the characteristics of light radiative transfer, such as scattering and absorption, from leaves or groups of plants. Mechanistic models provide a theoretical interpretation and are highly generalized. Recently, a few scholars have attempted to use mechanistic models to indirectly estimate nitrogen content [19]. These studies [19–21] primarily rely on physiological correlations between nitrogen content and parameters like chlorophyll, water content, and dry matter content. By estimating these biochemical parameters, nitrogen content can be indirectly inferred. However, these physiological relationships are not stable and are influenced by factors such as species, growing season, and external environment. Unlike the nonlinear relationships between chlorophyll and nitrogen, stable correlations between LNC and protein content can be found across numerous plant species in various seasons [16]. Indeed, due to relatively low protein content within leaves, as well as the fact that specific absorption features of protein can be partly overlapped by the absorption of water and other dry matter constituents, the spectroscopic estimation of protein becomes challenging [2]. Given the robustness and transferability of mechanistic models, Jacquemoud et al. [22] and Wang et al. [23] tried to update the physical models by calibrating the specific absorption coefficients of protein and other dry matter constituents. However, these 2 studies only considered the absorption features of proteins, lignin, and cellulose but rather not included the absorption of other nonstructural carbohydrates (e.g., sugars and starch), which represented about 25% of leaf dry mass per unit area (LMA) and thus led to significant uncertainties in the forward and inverse modeling [2].

Recently, Féret et al. [2] has made marked progress in estimating protein content and corresponding nitrogen content at the leaf scale. They optimized the specific absorption coefficients of nitrogen-associated proteins, particularly in the short-wave infrared region, based on the PROSPECT-D model [24]. This led to the development of the PROSPECT-PRO model, which successfully achieved the direct estimation of protein content and the corresponding LNC (through the conversion of protein content) at the leaf scale. Unlike previous models, the PROSPECT-PRO model separates dry matter content into nitrogen-related protein content and carbon-related content. It then optimizes the specific absorption coefficients of these components, providing a theoretical basis for the direct estimation of LNC.

In previous studies, the inversion of the PROSPECT model has been commonly conducted using leaf directional–hemispherical reflectance factor (DHRF) and transmittance factor (DHTF) spectra [2]. There are several challenges that affect the inversion accuracy, such as uncertainties in the simulated light absorption properties of molecules, errors during spectral measurements, and the spectral shadowing effects of other biochemicals on the spectral absorption features of target parameters [25]. As a result, these issues may contribute to the poor retrieval of certain constituents, such as LMA [26]. To improve the retrieval of target parameters, several studies have recommended the prior estimation of leaf structure parameter (N_struct_) and the preselection of sensitive spectral feature regions before the inversion of models [19,25,27–29]. Given the fact that the N_struct_ structure parameter dominates the scattering of photons within the leaf mesophyll, it substantially impacts leaf spectra across the entire spectral range from 400 to 2500 nm, especially in the near-infrared domains with low absorption. Thus, estimating N_struct_ prior to the model inversion is typically carried out at the initial stage of the retrieval process [25,27]. There are 3 main methods of estimating the N_struct_ parameter (Table 1), including the 3-wavelength inversion method, the sensitive spectral domain method, and the spectral ratio-index method. Among these methods, the latter one is simpler and has been demonstrated to improve PROSPECT inversion using reflectance or transmittance spectra alone [25]. However, these ratio-index studies have only evaluated their performance with DHRF or DHTF spectra, but not leaf bidirectional reflectance factor (BRF) spectra. Compared to DHRF spectra acquired by operating spectrometers equipped with integrating spheres, utilizing close-range imaging spectrometers or leaf clip-equipped spectrometers is more convenient and efficient to obtain BRF spectra, although BRF spectra are more susceptible to specular reflection on the leaf surface.

**Table 1.** Summary of the prior N_struct_ estimation using different approaches. Note: Cab, Car, Cw, Cm, and N_area_ represent chlorophyll, carotenoid, water, dry matter, and area-based nitrogen content, respectively. N_struct_ is the leaf structure parameter. BRF represents bidirectional reflectance factor spectra. BTF represents bidirectional transmittance factor spectra. DHRF represents directional–hemispherical reflectance factor spectra. DHTF represents directional–hemispherical transmittance factor spectra. Three-wavelength inversion method represents using 3 wavelengths to implement the inversion of N_struct_ by minimizing the merit function: they correspond to the maximum reflectance, the maximum transmittance, and the minimum absorptance in the NIR plateau (where the spectral response is more sensitive to N_struct_).

Target parameters	PROSPECT versions	Spectrum types	Methods for prior N_struct_ estimation	Spectral range used in the inversion of target parameters	References
Cab, Car, Cw, and Cm	PROSPECT-4 and 5	DHRF and DHTF	Three-wavelength inversion method using 3 wavelengths within 800–1300 nm	400–2500 nm	Féret et al. [27]
Cab, Cw, and Cm	PROSPECT-4	BRF	Minimizing its merit function using the full domain of 760–1300 nm	Cab: 400–760 nm; N_struct_: 760–1300 nm; Cw: 1400–1600, 1880–2100, and 2300–2500 nm; Cm: 1600–1800 and 2100–2400 nm based on the sensitivity analysis	Li and Wang [28]
N_area_, Cab, Cw, and Cm	PROSPECT-4	DHRF and DHTF	Three-wavelength inversion method using 3 wavelengths within 760–1300 nm	450–690 nm for Cab; 900–2500 nm for Cw and Cm following the prior information	Wang et al. [19]
N_struct_	PROSPECT-5	BRF and BTF	Three-wavelength inversion method using 3 wavelengths within 800–1300 nm	Three corresponding wavelengths within 800–1300 nm	Boren et al. [29]
Cab, Car, Cw, and Cm	PROSPECT-D	DHRF and DHTF	The linear models relating reflectance or transmittance ratios to N_struct_.	EWT and LMA: 1700– 2400 nm; Cab: 700– 720 nm; Car: 520–560 nm.	Spafford et al. [25]

In recent years, several studies [30–32] have utilized leaf BRF spectra to estimate leaf traits, including leaf pigment content, water content and dry matter content. However, to the best of our knowledge, few studies [32] have investigated the potential of using BRF spectra to estimate LNC of trees or other plants from the inversion of PROSPECT models. Specifically, previous studies had not considered the prior knowledge of N_struct_ information [30,31] or had performed the prior N_struct_ estimation using a standard ratio index developed based on leaf DHRF spectra [32] in the inversion of leaf traits. It remains uncertain whether LNC can be accurately extracted from BRF spectra with the corresponding prior N_struct_ estimation strategy, given the adverse impacts of the surface specular reflection and absorption from other biochemicals (e.g., water). In this study, we developed an advanced BRF spectra-based approach to prior estimate the N_struct_ structure parameter, by reducing the effects of specular reflection and enhancing LFC estimation in the shortwave-infrared spectrum region, using BRF spectra collected with leaf clip-equipped spectrometers. We examined the capability of this approach for nondestructively estimating LNC in leaves of *Ginkgo* trees and saplings. Specifically, we seek to answer 2 questions: (a) Whether and how can we develop spectral indices derived from leaf BRF data to improve prior N_struct_ estimation compared to the standard DHRF-based indices, by alleviating specular reflection effects? (ii) What are the optimal inversion methods and the most informative wavebands to retrieve LNC from leaf BRF data?

## Materials and Methods

### Study site description

#### Study area of *Ginkgo* trees

The sample leaves of *Ginkgo* trees were collected from middle-aged (23-year-old) and young-aged (14-year-old) *Ginkgo* Forest stands in Dongtai Forest (32°52′20.6″N, 120°49′32.2″E), which covers an area of 2239 ha. This is a subtropical planted forest in the coastal Jiangsu Province. The region has an average annual temperature of 14.6° and precipitation of 1050 mm. The *Ginkgo* Forest stands were initially planted with seeds of a main leaf-harvesting cultivar called “Taixing Dafozhi”. The 23-year-old plantations were grown as pure stands with a planting density of 4 × 4 m. In contrast, the 14-year-old plantations were planted in combination with *Euonymus alatus* as an agroforestry system, with a planting density of 2 × 8 m. Detailed information about this study area can be found in the study of Zhou et al. [33].

#### Study area of *Ginkgo* saplings

The experimental area for *Ginkgo* saplings is located in Baima Town, Lishui District, Nanjing, Jiangsu Province. The area experiences a subtropical monsoon climate, which lies within the transition zone between North-subtropical and Central-subtropical regions. The average annual temperature in this region is 15.4 °C, with an average annual sunshine duration of 2240 h and an average annual rainfall of 1106.5 mm. These favorable conditions make it a suitable location for planting *Ginkgo* saplings. The total experimental area dedicated to *Ginkgo* saplings is approximately 0.17 ha. The 2-year-old saplings were initially planted in 2020 with a height of around 0.5 m. The experimental area is divided into 3 rows, each consisting of 6 columns, resulting in a total of 18 plots. Each plot covers an area of 72 m^2^. The *Ginkgo* saplings were planted with the planting density of 0.6 × 1.0 m. Additionally, a 0.5-m-wide field was created between each plot to separate them from one another.

To study the effects of different fertilization levels on the growth of *Ginkgo* seedlings, various nitrogen treatments were applied to the 18 plots. These treatments included N0: 0 kg/hm^2^, N1: 225 kg/hm^2^, N2: 450 kg/hm^2^, N3: 675 kg/hm^2^, N4: 900 kg/hm^2^, and N5: 1125 kg/hm^2^. Each level of nitrogen fertilizer treatment was randomly replicated 3 times. Urea (46% N), calcium superphosphate (12% P_2_O_5_), and potassium chloride (60% K_2_O) were the fertilizers used in the experiment. Nitrogen fertilizer was applied 3 times per year. In March, 40% of the yearly application was placed in furrows. In May, another 40% was applied in shallow strips. Finally, in July, the remaining 20% of the yearly application was shallowly applied in strips. Phosphorus (6 kg/plot of calcium superphosphate) and potash fertilizers (1.68 kg/plot potassium chloride) were applied as a one-time basal fertilizer and incorporated into the furrows in March.

### Leaf reflectance spectra measurement

#### Leaf spectra measurement of *Ginkgo* trees

In this study, leaf samples were collected from 2 groups of trees: 23-year-old trees (28 trees with typical cone-shaped crowns) and 14-year-old trees (29 trees with typical cone-shaped crowns). Each group had 7 and 8 plots, respectively, with each plot covering an area of 400 m^2^. The leaf collection and leaf spectra measurement took place on 2020 August 23. In August (during the summer), the *Ginkgo* leaves in the study area were typically approaching maturity and exhibited a high photosynthetic activity with green color. With the use of a branch shear, 10 leaves around the same branch (south-facing side for holding similar lighting conditions) were collected from the upper canopy of each sampled tree. The collected leaves were carefully labeled, placed in sealed plastic bags, and stored in a cooling box with ice to maintain their freshness. In the laboratory, the leaf BRF spectra were recorded using leaf clip-equipped spectrometers (ASD FieldSpec 4 HR NG, Analytical Spectral Devices, Boulder, CO, USA). The leaf clip had a fixed illumination zenith angle of 12° and a fixed viewing zenith angle of 35° with the relative azimuth angle of 0°. It covered approximately a circular area with a radius of 0.5 cm. To minimize spectral noise, 10 leaves were scanned 3 times each, and their spectral measurements were averaged to produce a single spectrum that represented the leaf samples of each tree, following the strategy by Gara et al. [34].

#### Leaf spectra measurement of *Ginkgo* saplings

In this study, leaf samplings of 3-year-old *Ginkgo* saplings were conducted on 2021 July 7 and 2021 September 19, while leaf samplings of 4-year-old *Ginkgo* saplings were conducted on 2022 August 5 and 2022 September 26. The sampling dates were chosen to correspond with 2 important periods of fertilizer application, namely, the rapid growth period in spring (from late May to July, characterized by shoot branching and leaf expansion), and the later growth stage (i.e., plant maturity stage) in summer (from late July to September, marked by increased leaf thickness and accumulation of medicinal components), during which *Ginkgo* leaves remained highly physiologically active. Notably, leaf senescence did not start until at least mid-October. Although these dates fall after the astronomical spring (approximately March 20 to June 21), they were intentionally selected to capture nitrogen levels 1 to 2 months postfertilization, aligning with critical growth phases and fertilizer application periods. This approach enables us to observe the response of *Ginkgo* to nitrogen application and assess the impact of varying nitrogen levels on LNC during these critical stages.

In particular, 3 representative saplings were selected from individual experimental plots. For the 3-year-old sampled saplings, 12 representative south-facing leaves were selected only from the upper canopy of each sampled sapling. In contrast, regarding the 4-year-old sampled saplings, 12 representative south-facing leaves were selected from the upper and lower canopy of each sampled sapling, respectively. The collected leaves were carefully labeled, sealed in plastic bags, and stored in a cooler with ice to preserve freshness before being promptly transported to the lab for spectral analysis, with less than a 30-min interval between sampling and measurement. Then, these sampled leaves were subjected to spectra collection using leaf clip-equipped spectrometers (ASD FieldSpec 4 HR NG, Analytical Spectral Devices, Boulder, CO, USA). To minimize the spectral noise, 12 leaves from the upper canopy (or the lower canopy) of each sampled sapling were scanned 3 times each. The spectral measurements from these scans were then averaged to generate a single spectrum, representing the leaf samples of the upper canopy (or the lower canopy) for each sapling.

### Leaf biochemical parameter measurements

#### Leaf dry matter content and water content measurement

After collecting the leaf spectra, the leaf fresh weight (mg) and leaf area (LA, cm^2^) of each sampled leaf were measured immediately in the lab. In particular, LA was measured using a digital scanner with threshold segments [33]. The leaf was then dried in the oven at 80 °C for 48 h, and the dry weight (LDW, mg) was measured. The leaf fresh weight and LDW values were determined using an analytical balance with a precision of 0.0001 g. The LMA (also known as leaf dry matter content, mg/cm^2^) were determined according to the equation below:

$$\text{LMA}=\frac{\text{LDW}}{\text{LA}}$$ (1)

where LDW is the leaf dry weight (mg), and LA is the leaf surface area (cm^2^).

#### LNC and protein content measurement

After the LDW measurements, the dry leaf samples were finely ground and sieved (0.25 mm). Then, the mass-based LNC (LNC_mass_, mg/g) of the leaves was determined using the micro-Kjeldahl method [6]. The area-based LNC (LNC_area_, mg/cm^2^) was calculated as the product of LNC_mass_ and LMA. To convert LNC_area_ to leaf nitrogen-based protein content (Cp: expressed in protein mass per leaf unit area, mg/cm^2^), we applied the nitrogen-to-crude protein conversion factor of 4.43, as suggested by Yeoh and Wee [16] and Féret et al. [2]. The leaf carbon-based constituent content (CBC, mg/cm^2^), which includes lignin, cellulose, hemicellulose, and nonstructural carbohydrates such as sugars and starch, was determined as follows [2]:

$$\begin{array}{c} \text{CBC}\;\left(\text{mg}/{\text{cm}}^{2}\right)=\text{LMA}-\text{Cp}\\ =\text{LMA}-{\text{LNC}}_{\text{area}}\left(\text{mg}/{\text{cm}}^{2}\right)\times 4.43 \end{array}$$ (2)

### Prior estimation of the N_struct_ parameter

#### Reflectance spectra simulation

According to the hypothesis proposed by Merzlyak et al. [35] to develop a correcting factor for transmittance, the absorption can be negligible in the minimum absorption domains, such as the near-infrared regions (760 to 800 nm) with the incomplete collection of light photons from highly scattering tissues. Qiu et al. [36] revealed that the N_struct_ parameter strongly correlated with the ratio of the reflectance to transmittance at 800 nm. Based on these findings, Spafford et al. [25] proposed the equation that link N_struct_ to the ratio indices at the minimum absorption domains (e.g., 800 nm) as below:

$$\begin{array}{c} N_{\text{struct}}\propto \frac{R_{\text{minA}}}{T_{\text{minA}}}\approx \frac{R_{\text{minA}}}{1-R_{\text{minA}}}\approx \frac{1-T_{\text{minA}}}{T_{\text{minA}}} \end{array}$$ (3)

where $R_{\text{minA}}$ and $T_{\text{minA}}$ represent the reflectance and transmittance at the waveband with minimum absorption, respectively. Equation 3 provided the mechanism that allowed for estimating N_struct_ using the reflectance or transmittance alone. Spafford et al. [25] also performed the ratio-based prior N_struct_ estimation to enhance PROSPECT inversion with DHRF (or DHTF) spectra. As compared to the DHRF spectra, the leaf BRF spectra were easier and more efficient to obtain by operating close-range imaging spectrometers or leaf clip-equipped spectrometers as for a common practice. However, the potential of applying leaf BRF spectra in the prior N_struct_ estimation has not been fully exploited to enhance the PROSPECT model inversion yet, while the contribution of specular components was more pronounced in the BRF spectra compared to the DHRF spectra [33].

To analyze the specular effects on the relationships between N_struct_ and ratio index derived from the leaf BRF spectra, we conducted a reflectance simulation by incorporating the specular parameter *Bspec* (assumed to be wavelength-independent) into the PROSPECT-PRO model, following the approach used in the simplified PROCOSINE (sPROCOSINE) model [30,37]. As recommended by Spafford et al. [25], we generated 1000 leaf reflectance simulation spectra by adding *Bspec* to the PROSPECT-PRO model, with the intervals of leaf traits (N_struct_, Cab, Car, Cw, Cp, CBC, and Bspec) randomly sampled within the ranges specified in Table 2. These simulated reflectance spectra were calculated as below:

$$\bar{R_{\lambda }}=\tilde{R_{\lambda }}+\text{Bspec}$$ (4)

**Table 2.** The ranges of input parameters used for the model simulation. Note: LNC_area_ (mg/cm^2^) = Cp (mg/cm^2^) /4.43; LNC_mass_ (mg/g) = LNC_area_/LMA = LNC_area_/(Cp + CBC).

Parameters	Variable (unit)	Ranges
Leaf structure parameter	N_struct_ (unitless)	1.0–2.5
Leaf chlorophyll content	Cab (μg/cm^2^)	10–100
Leaf carotenoid content	Car (μg/cm^2^)	0.5–20
Leaf anthocyanin content	Canth (μg/cm^2^)	Fixed at 0
Leaf brown pigment content	Cbrown (μg/cm^2^)	Fixed at 0
Leaf water content	Cw (mg/cm^2^)	1–40
Leaf protein content	Cp (mg/cm^2^)	0–3
Leaf carbon-based constituent content	CBC (mg/cm^2^)	0–10
Leaf specular reflection factor	Bspec	0–0.3

where $\tilde{R_{\lambda }}$ is the simulated reflectance (i.e., simulated DHRF spectra) of the PROSPECT-PRO model, and $\bar{R_{\lambda }}$ is the updated simulated reflectance (i.e., simulated BRF spectra) after adding the specular constant parameter *Bspec*. To account for the realistic scenario of model inaccuracies and measurement noise, each simulated reflectance spectrum was combined with 0.3% random Gaussian noise [38,39]. Additionally, to clearly demonstrate the influence of specular factor *b* on the relationships of N_struct_ with standard and modified ratio indices, we generated the second simulated spectra dataset (only for the illustration and not for building the N_struct_ estimation models), with different N_struct_ (1.0, 1.3, 1.6, 1.9, 2.2, and 2.5) and *Bspec* values (0, 0.03, and 0.06), but fixed chlorophyll (45 μg/cm^2^), carotenoid (5 μg/cm^2^), anthocyanins (0 μg/cm^2^), brown pigments (0 μg/cm^2^), protein (0.8 mg/cm^2^), water (17 mg/cm^2^), and carbon-based constituent (8 mg/cm^2^) contents.

In particular, *Bspec* represents the difference ($b_{\lambda }$) in the specular reflection components of leaf surface between DHRF spectra and BRF spectra, when assuming this difference is a constant [30,33,37,40]. The connection between DHRF and BRF can be defined simply as below:

$$Bs\mathrm{pec}=b_{\lambda }={\mathrm{BRF}}_{\lambda }-{\text{DHRF}}_{\lambda }={\mathrm{BRF}}_{\lambda }^{\text{Surf}}-{\text{DHRF}}_{\lambda }^{\;\text{Surf}}$$ (5)

#### Modified ratio indices for the prior N_struct_ estimation

According to the findings in Spafford et al. [25], the ratio index derived from the DHRF spectra of optimal bands (the minimum absorption domain of 800 nm for the range of 400 to 800 nm; the minimum absorption domain of 1131 nm for the range of 400 to 2500 nm) showed a strong correlation with N_struct_.

$$N_{\text{struct}}\propto \frac{R_{\text{minA}}}{T_{\text{minA}}}\approx \frac{R_{\text{minA}}}{1-R_{\text{minA}}}=\frac{{\text{DHRF}}_{800}}{1-{\text{DHRF}}_{800}}$$ (6)

$$N_{\text{struct}}\propto \frac{R_{\text{minA}}}{T_{\text{minA}}}\approx \frac{R_{\text{minA}}}{1-R_{\text{minA}}}=\frac{{\text{DHRF}}_{1131}}{1-{\text{DHRF}}_{1131}}$$ (7)

However, compared to DHRF spectra, the BRF spectra are more sensitive to the specular reflection. While adding a constant of specular factor *b* to DHRF_λ_ in Eqs. 6 and 7, the original DHRF_λ_/(1 − DHRF_λ_) does not equal to (DHRF_λ_ + *b*) /(1 − (DHRF_λ_ + *b*)) if *b* ≠ 0. This implies that the standard ratio indices of prior N_struct_ estimation can be sensitive to the specular reflection.

To mitigate the sensitivity of ratio index to specular reflection, we followed the strategy used by Sims and Gamon [41] and Li et al. [30], to assume the reflectance of 445 nm (R_445_) and 1925 nm (R_1925_) as the specular reflectance (i.e., the constant of specular factor *b*) in the visible and near-infrared (VNIR) region (400 to 800 nm) and the shortwave infrared (SWIR) region (1000 to 2500 nm), respectively. The linear relationships between N_struct_ and standard or modified ratio indices are listed in Table 3. Thus, Eqs. 6 and 7 can be revised as below, by removing the specular reflection in BRF spectra based on the connection between BRF and DHRF (Eq. 5):

$$\begin{array}{c} N_{\text{struct}}\propto \frac{{\text{DHRF}}_{800}}{1-{\text{DHRF}}_{800}}\\ =\frac{{\text{BRF}}_{800}-b_{\lambda }}{1-\left({\text{BRF}}_{800}-b_{\lambda }\right)}\approx \frac{{\text{BRF}}_{800}-{\text{BRF}}_{445}}{1-\left({\text{BRF}}_{800}-{\text{BRF}}_{445}\right)} \end{array}$$ (8)

$$\begin{array}{c} N_{\text{struct}}\propto \frac{{\text{DHRF}}_{1131}}{1-{\text{DHRF}}_{1131}}\\ =\frac{{\text{BRF}}_{1131}-b_{\lambda }}{1-\left({\text{BRF}}_{1131}-b_{\lambda }\right)}\approx \frac{{\text{BRF}}_{1131}-{\text{BRF}}_{1925}}{1-\left({\text{BRF}}_{1131}-{\text{BRF}}_{1925}\right)} \end{array}$$ (9)

**Table 3.** The prior estimation of N_struct_ using different strategies. Note: No_Prior represents the approach that does not perform the prior N_struct_ estimation. Prior_800 represents the approach that applies the standard ratio indices of 800-nm reflectance (R_800_) to the prior N_struct_ estimation. Prior_1131 represents the approach that applies the standard ratio indices of 1131-nm reflectance (R_1131_) to the prior N_struct_ estimation. mPrior_800 represents the approach that applies the modified ratio indices of 800-nm reflectance (R_800_) to the prior N_struct_ estimation, by removing the 445-nm reflectance represented as the specular reflection in the VNIR region. mPrior_1131 or mPrior_1365 represents the approach that applies the modified ratio indices of 1131-nm reflectance (R_1131_) or 1365-nm reflectance (R_1365_) to the prior N_struct_ estimation, by removing the 1925-nm reflectance represented as the specular reflection in the SWIR region.

Strategy	Ratio indices	The N_struct_ estimation model	References
No_Prior	-	-	-
Prior_800	$\frac{R_{800}}{1-R_{800}}$	${\text{N}}_{\text{struct}}=1.724\times \frac{R_{800}}{1-R_{800}}+0.0795$	Spafford et al. [25]
Prior_1131	$\frac{R_{1131}}{1-R_{1131}}$	${\text{N}}_{\text{struct}}=1.830\times \frac{R_{1131}}{1-R_{1131}}+0.0711$	Spafford et al. [25]
mPrior_800	$\frac{R_{800}-R_{445}}{1-\left(R_{800}-R_{445}\right)}$	${\text{N}}_{\text{struct}}=1.906\times \frac{R_{800}-R_{445}}{1-\left(R_{800}-R_{445}\right)}$ + 0.1658	Developed in this study
mPrior_1131	$\frac{R_{1131}-R_{1925}}{1-\left(R_{1131}-R_{1925}\right)}$	${\text{N}}_{\text{struct}}=1.6183\times \frac{R_{1131}-R_{1925}}{1-\left(R_{1131}-R_{1925}\right)}$ + 0.5106	Developed in this study
mPrior_1365	$\frac{R_{1365}-R_{1925}}{1-\left(R_{1074}-R_{1925}\right)}$	${\text{N}}_{\text{struct}}=2.1829\times \frac{R_{1365}-R_{1925}}{1-\left(R_{1074}-R_{1925}\right)}$ + 0.4956	Developed in this study

To further explore the relationships between N_struct_ and the modified ratio indices in the SWIR regions, the calculation of the ratio-index formulation was performed (Eq. 10), based on the full combinations of 2 wavebands at λ1 and λ2 within 1000 to 1400 nm, which had a higher sensitivity to N_struct_ than other SWIR wavelengths (as indicated in Fig. 2 from Spafford et al. [25]). In particular, the most sensitive waveband combinations were obtained according to the highest squared Pearson’s correlation coefficients (*r*^2^) in contour maps between N_struct_ and ratio indices [33]. Additionally, these 2 selected bands required the minimum interval larger than 10 nm to enhance the robustness of N_struct_ estimation models, by avoiding the spectral noise that may occur for close wavelengths [33,39].

$$N_{\text{struct}}\propto \frac{{\text{BRF}}_{\lambda 1}-{\text{BRF}}_{1925}}{1-\left({\text{BRF}}_{\lambda 2}-{\text{BRF}}_{1925}\right)}$$ (10)

The contour map of *r*^2^ between Ns and the modified ratio indices is displayed in Fig. S1, with the use of 1925 nm as the reference waveband. The higher *r*^2^ areas (*r*^2^ > 0.73) were obtained taking λ1 in the region of 1310 to 1380 nm and λ2 in the region of 1000 to 1135 nm. In particular, the optimal waveband combinations of modified ratio indices were obtained as mPrior_1365 $(\;\left[{\text{BRF}}_{1365}-{\text{BRF}}_{1925}\right]$/$\left[1-\left({\text{BRF}}_{1074}-{\text{BRF}}_{1925}\right)\right]\;)$, with the highest *r*^2^ with N_struct_. We used this index to compare with the existing standard ratio index in relating with N_struct_ across the simulated spectra encompassed numerous physiological conditions. Additionally, the combinations of choosing λ1 from 1150 to 1300 nm and λ2 from 1150 to 1400 nm also yields a high *r*^2^ (*r*^2^ = 0.70 to 0.72)*.*

### PROSPECT-PRO model inversion

**Fig. 1.**
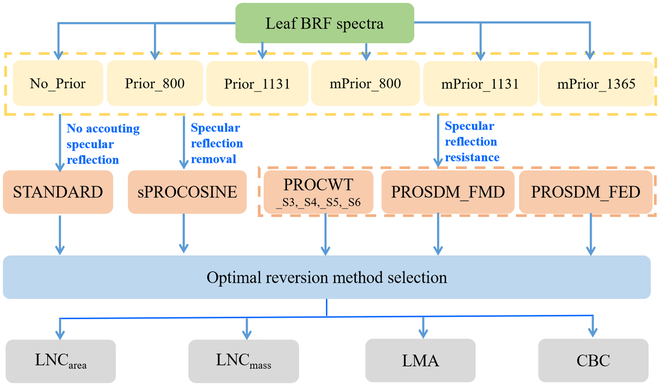
The flowchart for the PROSPECT-PRO inversion procedures.

In this study, the above 6 prior N_struct_ estimation strategies were combined with 8 inversion methods to invert PROSPECT-PRO for retrieving CBC, LMA, LNC_area_, and LNC_mass_, from the BRF spectra in the *Ginkgo* tree and sapling datasets. These 48 approaches (6 prior N_struct_ estimation strategies × 8 inversion methods) had the same input of leaf BRF spectra but differed in the prior N_struct_ estimation and the mathematical optimization of the inversion merit function (Fig. 1).

In particular, 6 prior N_struct_ estimation strategies included No_Prior (i.e., no prior N_struct_ estimation performed), Prior_800 (i.e., prior N_struct_ estimation using the standard ratio indices of 800-nm reflectance), Prior_1131 (i.e., prior N_struct_ estimation using the standard ratio indices of 1131-nm reflectance), mPrior_800 (i.e., prior N_struct_ estimation using the modified ratio indices of 800-nm reflectance), mPrior_1131 (i.e., prior N_struct_ estimation using the modified ratio indices of 1131-nm reflectance), and mPrior_1365 (i.e., prior N_struct_ estimation using the modified ratio indices of 1365-nm reflectance).

The 8 inversion methods included the STANDARD (i.e., the standard inversion as the benchmark method), sPROCOSINE (i.e., the simplified PROCOSINE method), PROSDM_FED (i.e., PROSPECT inversion with the spectral first-order derivative and the similarity metric of Euclidean distance), PROSDM_FMD (i.e., PROSPECT inversion with the spectral first-order derivative and the similarity metric of Manhattan distance), PROCWT_S3, PROCWT_S4, PROCWT_S5, and PROCWT_S6 (i.e., coupling PROSPECT with continuous wavelet transform using scale 3 to 6).

Specifically, when performing PROSPECT-PRO inversion, the STANDARD method did not account for the leaf specular effects. In contrast, sPROCOSINE, PROCWT, PROSDM_FMD, and PROSDM_FED were able to reduce adverse effects of leaf specular reflection to varying extents, employing 2 different types of specular disturbance alleviation techniques [33,42]. The sPROCOSINE method belongs to the type of specular reflection removal, in which the leaf specular reflectance (or specular effects) should be estimated (such as the specular factor *b* in sPROCOSINE) and then removed from the original reflectance spectra. The PROCWT [30], PROSDM_FMD, and PROSDM_EFD methods [31] belong to the type of specular reflection resistance, which does not estimate actual specular effects but rather proposes operation methods (such as derivative-related operations) to partly reduce the impact of specular reflection. In particular, previous studies have confirmed that PROCWT exhibits properties of linear additivity and convergence [30], making it suitable for eliminating the additive effect on BRF spectra caused by the specular reflection from leaf surfaces. More detailed information about these methods can be found in the referenced studies [30,31]. Among these methods tested, several aligned well with our objective of improving LNC prediction through accurate prior estimation of N_struct_ using modified ratios. Based on this evaluation, the selection of optimal inversion approaches was achieved, by combing the most effective inversion method (e.g., PROCWT_S3) with the N_struct_ prior estimation strategy, allowing for further analysis in the spectral domain selection.

### The spectral domain selection for the PROSPECT-PRO inversion

In this study, we used the leaf reflectance spectra within the range of 1400 to 2399 nm to retrieve leaf nitrogen-related traits, following the recommend spectral domains for PROSPECT-PRO inversion in the study of Féret et al. [2]. In particular, the retrieval of LNC_area_ was accomplished using the conversion factor derived from the retrieved leaf protein content (the result of the retrieval of Cp was not shown for their consistency to LNC_area_). The retrieval of LNC_mass_ was done by calculating the ratio of the estimated LNC_area_ to the estimated LMA, when performing PROSPECT-PRO inversion simultaneously. In addition, we also attempted to optimize the LNC_mass_ retrieval by combining the optimal LNC_area_ retrieval with the best LMA retrieval from different inversion approaches. Furthermore, to determine the optimal spectral domains for the retrieval of LNC_area_ and LNC_mass_, we further applied the technique of sequential forward feature selection (SFS). Given the fact that the iterative optimization process in the SFS require extensive computation time (approximately 7 to 10 d for each examined approach) across all the datasets, it is difficult to perform SFS for all the examined approaches. Thus, we made a compromise to conduct SFS only for the optimal inversion approaches, which ranked top 25% in the retrieval of all the examined traits (i.e., LMA, CBC, and LNC_area_). Specifically, the optimal spectral domains were selected from 50 spectral features (each feature includes 20 wavebands with the 1-nm spectral sampling) in the domains of 1400 to 2399 nm, as the procedure suggested by Féret et al. [2] and Wang et al. [32].

### The validation of the PROSPECT-PRO model inversion

In this study, we conducted the LNC retrievals using measured reflectance data and the PROSPECT-PRO RTM. Notably, this approach does not require building regression models based on spectral data and LNC values from leaf samples. Instead, we evaluated the inversion performance, by comparing the measured LNC values from all collected leaf samples in ground-truth datasets with their corresponding estimated LNC values, obtained through the PROSPECT-PRO model during the inversion process. This approach aligns with previous studies that demonstrate the effectiveness of direct inversion methods for accurately estimating LNC without relying on calibration samples [2,32].

The mathematical optimization process for all model inversions and the optimal spectral subdomains selections were implemented with the “CONSTRAINED_MIN” function in IDL 8.5 (Exelis Visual Information Solutions, Boulder, CO, USA). To compare the PROSPECT-PRO model inversion performance between different inversion approaches, the coefficient of determination (*R*^2^) and the normalized root mean square error (NRMSE) was chosen to evaluate the robustness of the retrieval ability. The equations were listed as below:

$$R^{2}=1-\frac{\sum_{i=1}^{n}{\left({\hat{y}}_{i}-y_{i}\right)}^{2}}{\sum_{i=1}^{n}{\left(\bar{y}-y_{i}\right)}^{2}}$$ (11)

$$\text{NRMSE}=\frac{1}{\bar{y}}\sqrt{\frac{1}{n}\cdot \sum_{i=1}^{n}{\left({\hat{y}}_{i}-y_{i}\right)}^{2}}$$ (12)

where $y_{i}$ is the measured value and ${\hat{y}}_{i}$ is the estimated value for a leaf *i*, $\bar{y}$ is the mean value of measurements for all leaf samples, and n is the number of all leaf samples.

## Results

### Descriptive statistics of leaf constituents

**Fig. 2.**
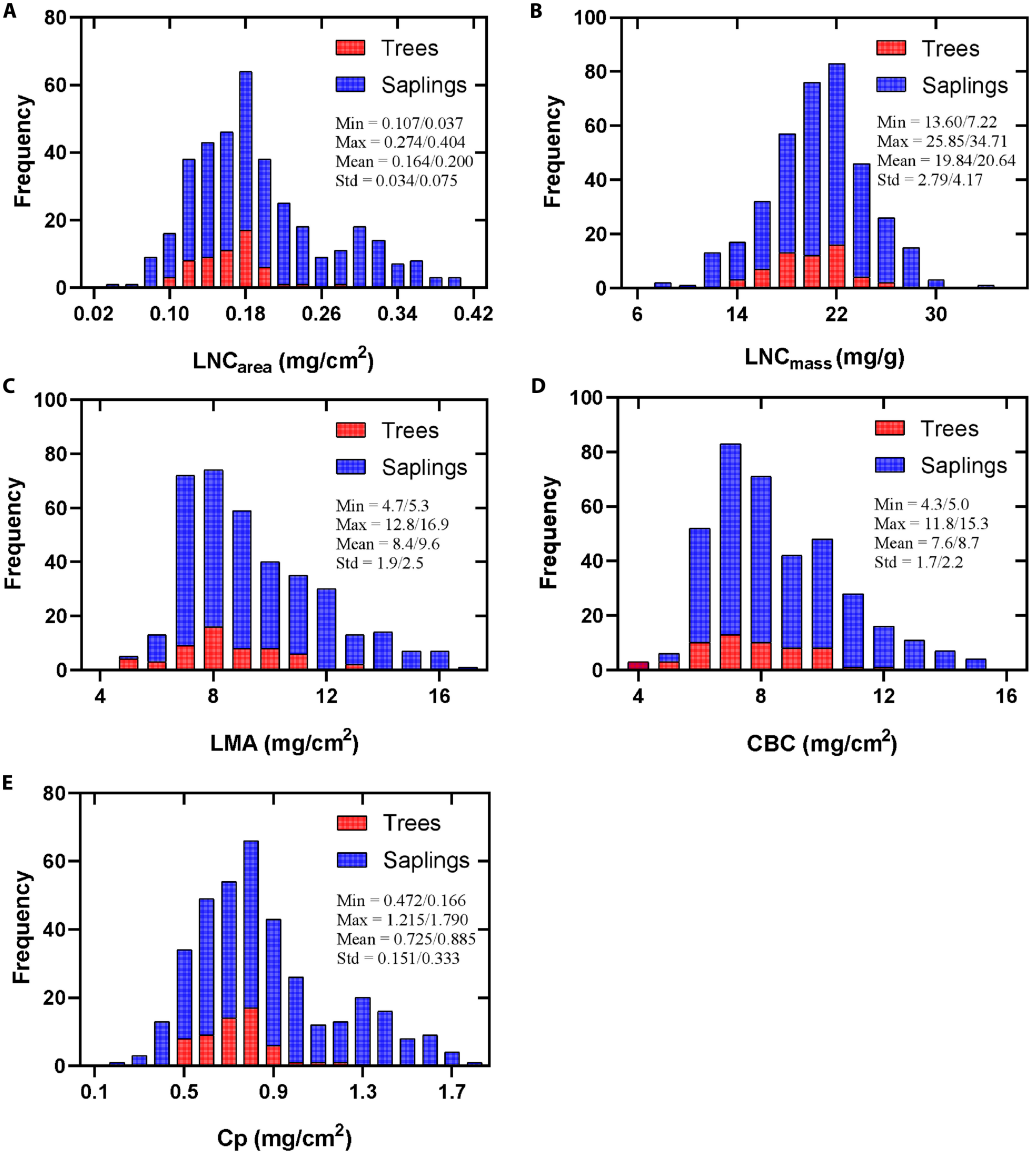
Stacked frequency distributions of (A) LNC_area_, (B) LNC_mass_, (C) LMA, (D) CBC, and (E) Cp for the leaf samples of *Ginkgo* trees and Saplings from all sampling times. The statistic information in each distribution plot is displayed before and after the slash (/), respectively.

Figure 2 shows the stacked frequency distribution of LNC_area_, LNC_mass_, LMA, CBC, and Cp for leaf samples of *Ginkgo* trees and saplings. Notably, the sapling samples exhibited relatively higher values of LNC_area_, LMA, and Cp compared to the tree samples. However, LNC_mass_ and CBC values were relatively similar between saplings and trees. Additionally, the range of all examined biochemical constituents was larger for the sapling samples compared to the tree samples.

**Fig. 3.**
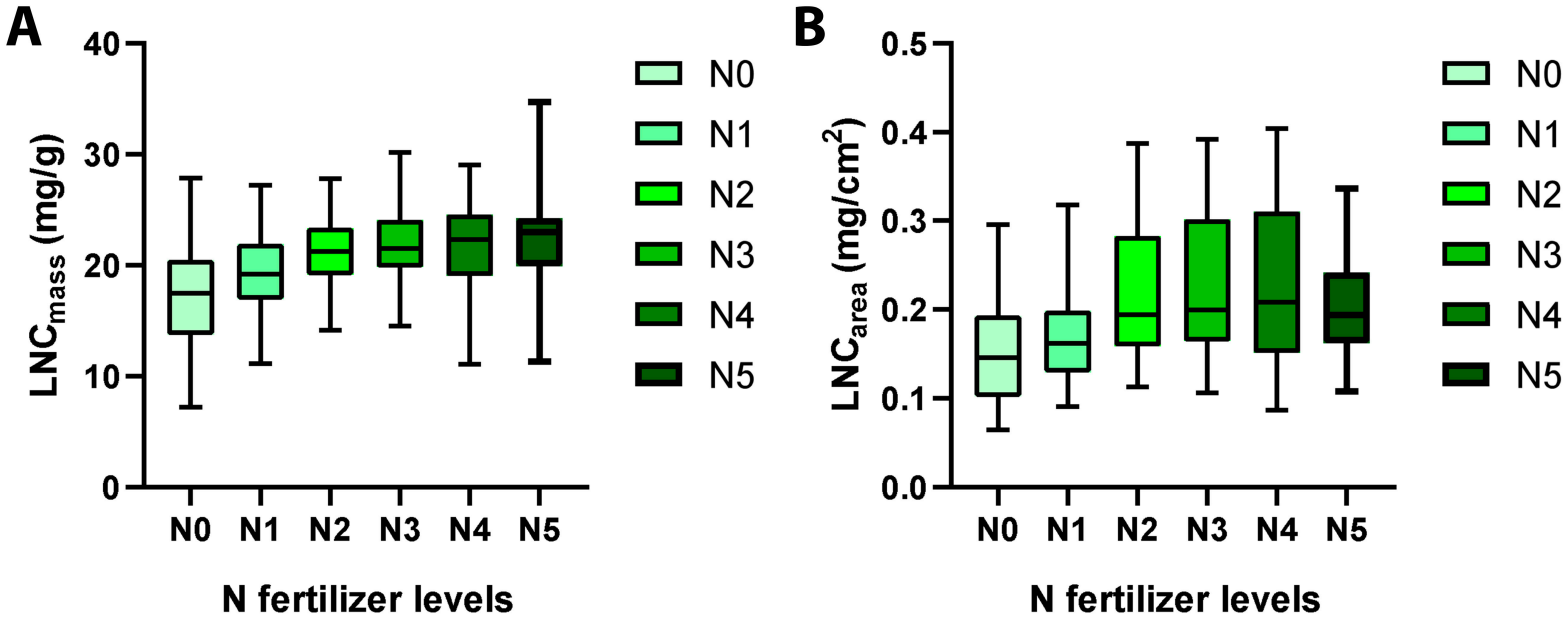
The boxplot of (A) LNC_mass_ and (B) LNC_area_ for the leaf samples of *Ginkgo* saplings from all sampling times under different nitrogen (N) fertilizer levels. N0 to N5 represent different nitrogen fertilizer treatments, which included N0: 0 kg/hm^2^, N1: 225 kg/hm^2^, N2: 450 kg/hm^2^, N3: 675 kg/hm^2^, N4: 900 kg/hm^2^, and N5: 1125 kg/hm^2^. R1 to R3 represent 3 nitrogen fertilizer treatment repetitions.

Figure 3 displays the boxplot of LNC_mass_ and LNC_area_ for the leaf samples of saplings under different nitrogen (N) fertilizer levels. The data represent samples collected at all sampling times (from July to September) during this study, which may account for the observed variability in LNC. It shows that both LNC_mass_ and LNC_area_ increased as the nitrogen fertilizer levels raised, reaching a peak at the N3 level (675 kg/hm^2^). Subsequently, the values remained stable or slightly decreased from N3 (675 kg/hm^2^) to N5 (1125 kg/hm^2^) levels. Differences in nitrogen levels across all sampling times, combined with variations in sampling locations (upper and lower canopy for 4-year-old saplings), likely contributed to the relatively low nitrogen content and high variability.

### Comparison of the leaf spectral characteristics under different LNC levels

**Fig. 4.**
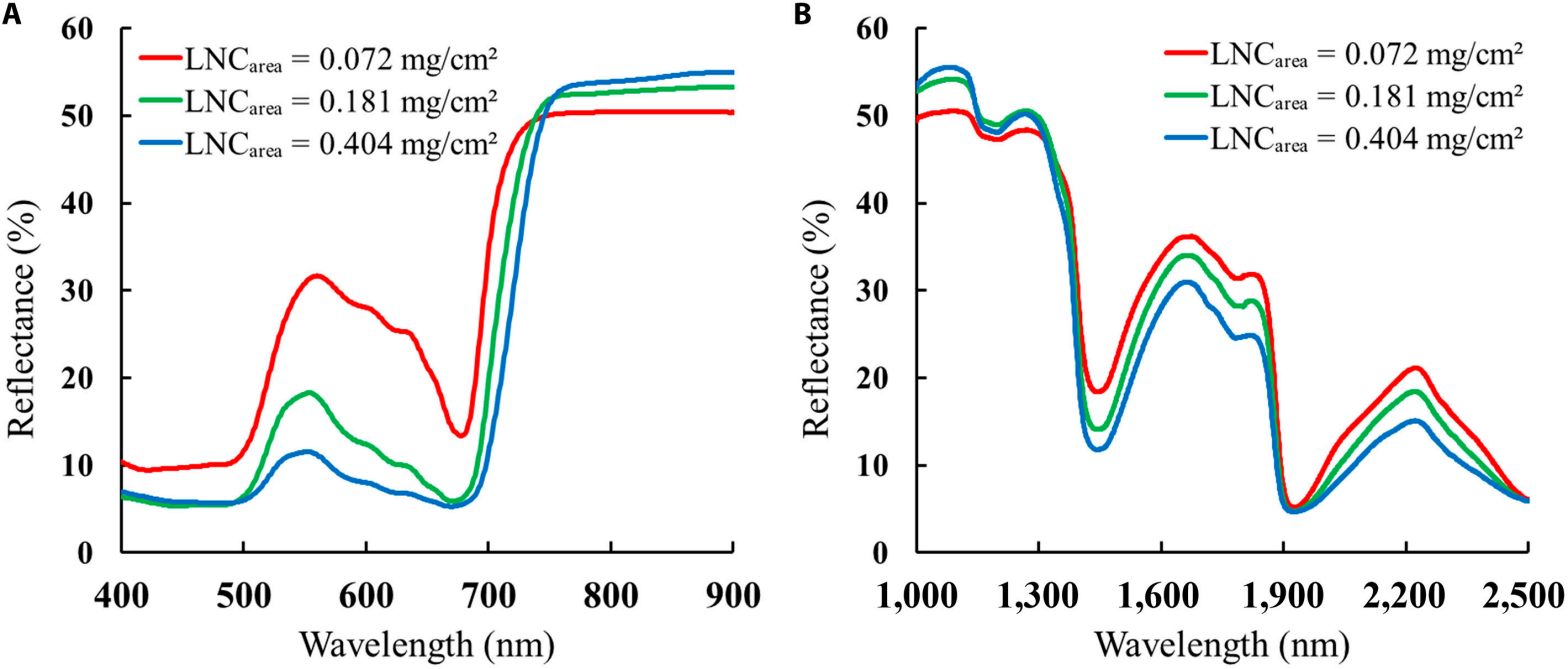
The measured reflectance spectra of leaf samples with low-level (0.072 mg/cm^2^), medium-level (0.181 mg/cm^2^), and high-level (0.404 mg/cm^2^) LNC_area_ values in the spectral ranges of (A) 400 to 900 nm and (B) 1000 to 2500 nm.

As illustrated in Fig. 4, when the LNC_area_ increases, there is a gradual decline in the reflectance of the visible (400 to 700 nm) and the short-wave infrared regions (1400 to 2500 nm) but an increase in the reflectance of the near-infrared band region (750 to 1400 nm).

### The sensitivity of simulated SIs~N_struct_ relationships to the specular reflectance

**Fig. 5.**
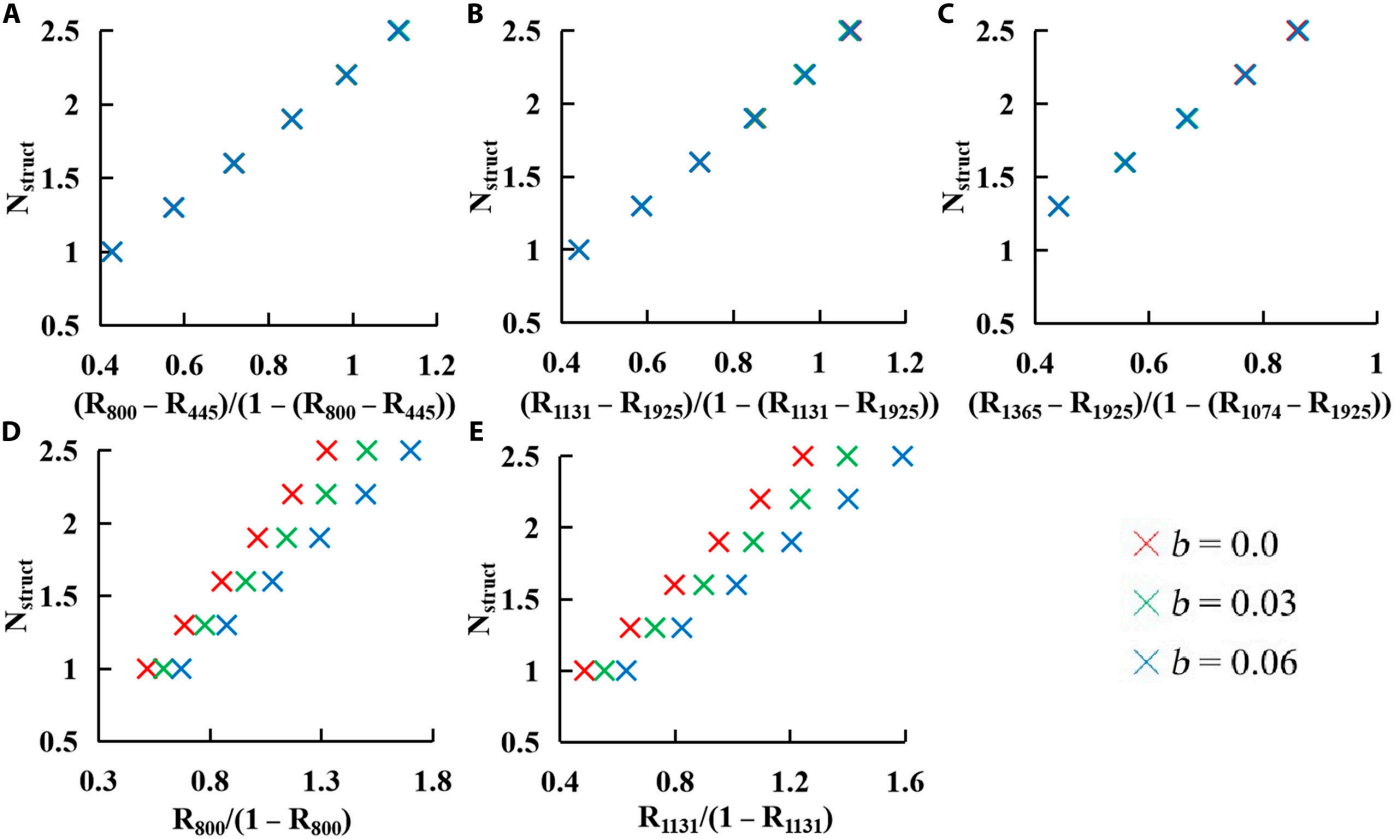
The linear relationships between N_struct_ and the modified ratio indices (A: (R_800_ − R_445_)/(1 − (R_800_ − R_445_)); B: (R_1131_ − R_1925_)/(1 − (R_1131_ − R_1925_); and C: (R_1365_ − R_1925_)/(1 − (R_1074_ − R_1925_)) and standard ratio indices (D: R_800_/(1 − R_800_) and E: R_1131_/(1 − R_1925_)) derived from the simulated reflectance by adding *Bspec* into the PROSPECT-PRO model.

Figure 5 shows the influence of specular reflectance (*b*) on the simulated relationships of N_struct_ with the standard and modified ratio indices. Generally, the standard ratio indices (Prior_800: R_800_/(1 − R_800_); Prior_1131: R_1131_/(1 − R_1131_)) were affected by the specular reflection, whereas the modifies ratio indices (mPrior_800: (R_800_-R_445_)/(1 − (R_800_-R_445_); mPrior_1131: (R_1131_-R_1925_)/(1 − (R_1131_-R_1925_); mPrior_1365: (R_1365_-R_1925_)/(1 − (R_1074_-R_1925_) exhibited no sensitivity to the specular reflectance factor *b*. Specifically, Prior_800 and Prior_1131 showed a rise tendency as the specular reflectance increased. Additionally, these standard index values turned to be more dispersed between 3 specular reflectance levels for the simulation spectra with higher N_struct_ values.

### The correlation analysis for different prior N_struct_ estimation indices

**Fig. 6.**
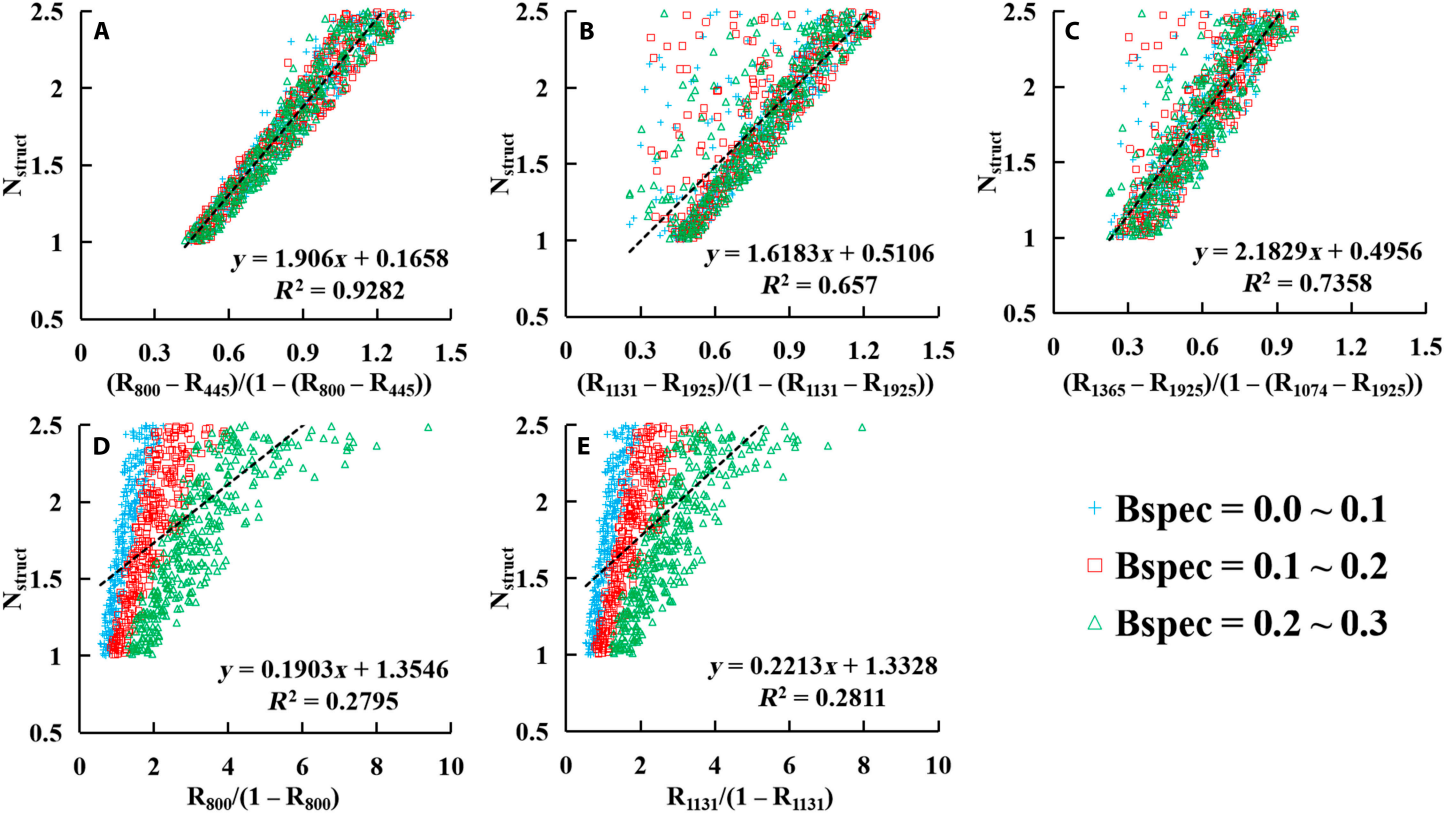
The linear relationships between N_struct_ and the modified ratio indices (A: (R_800_ − R_445_)/(1 − (R_800_ − R_445_)); B: (R_1131_ − R_1925_)/(1 − (R_1131_ − R_1925_); and C: (R_1365_ − R_1925_)/(1 − (R_1074_ − R_1925_)) and the standard ratio indices (D: R_800_/(1 − R_800_) and E: R_1131_/(1 − R_1131_)) derived from the simulated reflectance by adding *Bspec* into the PROSPECT-PRO model.

Figure 6 shows the linear relationships between N_struct_ and the standard or modified ratio indices derived from the simulated reflectance spectra in Table 2, with the addition of *Bspec* into the PROSPECT-PRO model. The standard ratio indices (Prior_800: R_800_/(1 − R_800_); Prior_1131: R_1131_/(1 − R_1131_)) had weak sensitivity to N_struct_ (*R*^2^ = 0.28, *P* < 0.0001), whereas the modified ratio indices (mPrior_800: (R_800_ − R_445_)/(1 − (R_800_ − R_445_); mPrior_1131: (R_1131_ − R_1925_)/(1 − (R_1131_ − R_1925_); mPrior_1365: (R_1365_ − R_1925_)/(1 − (R_1074_ − R_1925_)) showed significant correlation with N_struct_ (*R*^2^ = 0.93, *P* < 0.0001 for mPrior_800; *R*^2^ = 0.66, *P* < 0.0001 for mPrior_1131; *R*^2^ = 0.74, *P* < 0.0001 for mPrior_1365). In particular, the Prior_800 and Prior_1131 values exhibited more dispersed tendency between 3 different *Bspec* levels (*Bspec* = 0.0 to 0.1; *Bspec* = 0.1 to 0.2; *Bspec* = 0.2 to 0.3) for the simulated spectra with higher N_struct_ values (Fig. 6A and B). Additionally, the N_struct_~Prior_800 or N_struct_~Prior_1131 relationships for the simulated spectra under the low *Bspec* level are stronger and more concentrate than those under the high *Bspec* level.

### Model inversions using different strategies

#### Retrieval of LMA and CBC

Table 4 illustrates the accuracy of PROSPECT-PRO inversions for retrieving LMA and CBC, using 6 prior N_struct_ estimation strategies (No_Prior, Prior_800, Prior_1131, mPrior_800, mPrior_1131, and mPrior_1365), and 8 inversion methods (PROCWT with scales ranging from 3 to 6, PROSDM_FED, PROSDM_FMD, STANDARD, and sPROCOSINE).

**Table 4.** The *R*^2^ and NRMSE values for the retrieval of LMA and CBC using different approaches


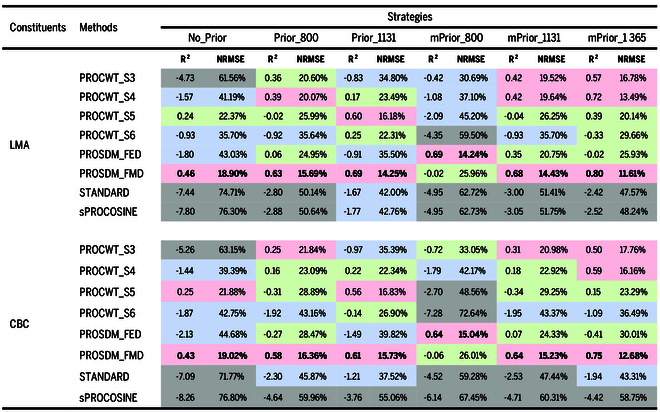

Note: The *R*^2^ and NRMSE values in bold indicate the highest and the lowest values by column, respectively. Negative *R*^2^ values indicate poor retrieval performance and transferability. The background colors for NRMSE values show the rank levels of inversion performance, ranging from light red (top 25%), green, blue, to gray (bottom 25%).

**Fig. 7.**
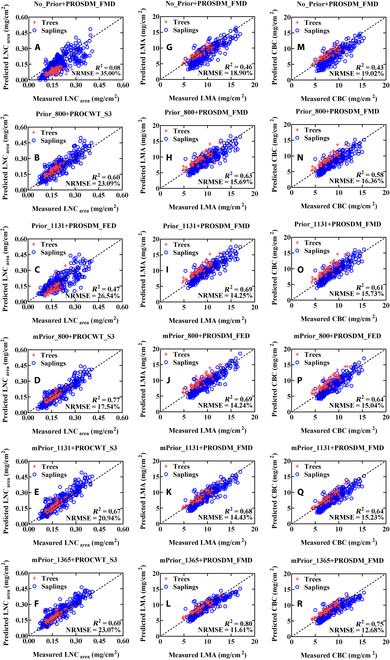
The predicted LNC_area_ (the left column), LMA (the middle column), and CBC (the right column) for the PROSPECT-PRO inversions from (A, G, and M) No_prior, (B, H, and N) Prior_800, (C, I, and O) Prior_1131, (D, J, and P) mPrior_800, (E, K, and Q) mPrior_1131, and (F, L, and R) mPrior_1365 with the corresponding best-performed inversion methods (are displayed after the plus sign “+”).

In regard to the prior N_struct_ estimation strategies, utilizing prior N_struct_ estimation with both standard and modified ratio indices resulted in enhanced inversion performance compared to the approach without them. Additionally, the use of modified ratio indices further improved the inversion accuracy over preexisting standard ratio indices (Fig. 7), particularly with SWIR region based modified ratio indices (mPrior_1365 for LMA and CBC) achieving the best performance for retrieving all 3 examined traits (LMA and CBC).

Concerning the inversion methods, the PROSDM_FMD-based retrieval of LMA (*R*^2^ = 0.80 and NRMSE = 11.61% for the best-performed case with mPrior_1365 combined PROSDM_FMD), and CBC (*R*^2^ = 0.75 and NRMSE = 12.68% for the best-performed case with mPrior_1365 combined PROSDM_FMD) generally outperformed other inversion methods (Fig. 7). Additionally, low-scale PROCWT-type methods (i.e., PROCWT-S3 and PROCWT-S4) combined mPrior_1131 or mPrior_1365 also yielded preferable performance (with the rank level of top 25%) in predicting LMA and CBC.

#### Retrieval of LNC_area_ and LNC_mass_

Table 5 shows the retrieval accuracies of LNC_area_ and LNC_mass_ using various approaches. In general, the prediction performance of LNC_area_ and LNC_mass_ was inferior compared to LMA and CBC. In terms of the prior N_struct_ estimation strategies for retrieving LNC_area_, the modified ratio indices outperformed the standard ratio indices and the strategy of no prior N_struct_ estimation. Regarding the performance of different inversion methods (Fig. 7), PROCWT_S3 yielded superior results, especially when combined with modified ratio indices (i.e., mPrior_800, mPrior_1131, and mPrior_1365) resulting in the best prediction performance (*R*^2^ = 0.60 to 0.77; NRMSE = 17.54 to 23.07%). Notably, the prediction accuracy of LNC_mass_ was not satisfactory with each single approach, as evidenced most NRMSE values exceeding 30% with negative *R*^2^ values when calculating the ratio of retrieved LNC_area_ to LMA simultaneously. Alternatively, indirectly estimating LNC_mass_ (*R*^2^ = 0.10 and NRMSE = 18.46%) was possible based on using the best case for the predicted LNC_area_ (mPrior_800 combined with PROCWT_S3) and LMA (mPrior_1365 combined with PROSDM_FMD) individually.

**Table 5.** The *R*^2^ and NRMSE values for the retrieval of LNC_area_ and LNC_mass_ using different approaches


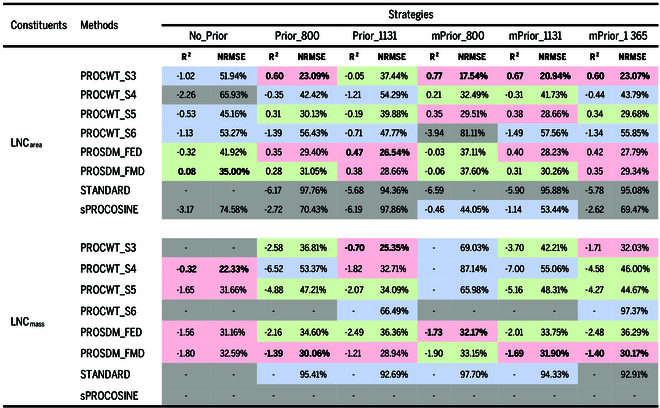

Note: The *R*^2^ and NRMSE values in bold indicate the highest and the lowest values by column, respectively. The symbol “-” represents NRMSE values greater than 100% or negative *R*^2^ values lower than −10 with no statistical significance. Negative *R*^2^ values indicate poor retrieval performance and transferability. The predicted LNC_mass_ values in Table 5 were calculated from the ratio of the predicted LNC_area_ to LMA with each single approach simultaneously. The background colors for NRMSE values show the rank levels of inversion performance, ranging from light red (top 25%), green, blue, to gray (bottom 25%).

#### The optimal spectral domains for retrieving LNC_area_ and LNC_mass_

**Fig. 8.**
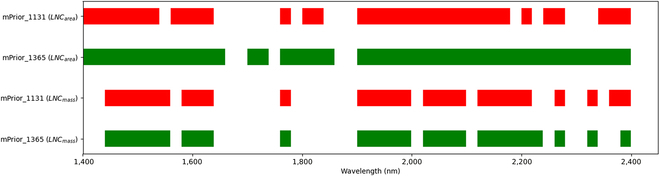
The optimal spectral domains for estimating LNC_area_ and LNC_mass_ using the SFS optimization procedure and the approach of PROCWT_S3 combined with mPrior_1131 (in red color) or mPrior_1365 (in green color).

Given the fact that the inversion approach of combining PROCWT_S3 with mPrior_1131 (or mPrior_1365) achieved the retrieval performance ranked top 25% for all examined traits (Tables 4 and 5), these 2 approaches were used with the SFS technique in the determination of optimal spectral domains for retrieving LNC_area_ and LNC_mass_ (Fig. 8). The optimal spectral domains to predict LNC_area_ or LNC_mass_ were mostly overlapped for using mPrior_1131 and mPrior_1365 combined with PROCWT_S3. Generally, the optimal regions to estimate LNC_area_ and LNC_mass_ for mPrior_1131 and mPrior_1365 had 5 common isolated spectral segments (in which at least 3 contiguous optimal spectral features) of 1440 to 1539 nm, 1580 to 1639 nm, 1900 to 1999 nm, 2020 to 2099 nm, and 2120 to 2179 nm. In particular, when estimating LNC_area_, the optimal domains for mPrior_1131 and mPrior_1365 shared 4 main isolated spectral domains, spanning the ranges of 1400 to 1539 nm, 1560 to 1639 nm, 1900 to 2179 nm, and 2340 to 2399 nm. In terms of LNC_mass_, both mPrior_1131 and mPrior_1365 shared 5 main common isolated spectral regions of 1440 to 1559 nm, 1580 to 1639 nm, 1900 to 1999 nm, 2020 to 2099 nm, and 2120 to 2219 nm.

**Fig. 9.**
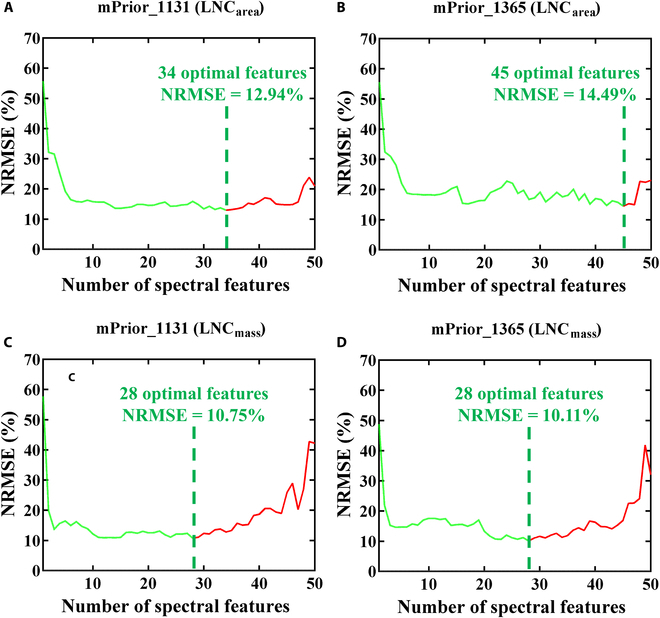
The evolved NRMSE (%) values for estimating LNC_area_ (A and B) or LNC_mass_ (C and D) with an increasing number of spectral features using the SFS optimization procedure and the approach of PROCWT_S3 combined with mPrior_1131 or mPrior_1365.

Figure 9 depicts the evolution of NRMSE (%) values for estimating LNC_area_ and LNC_mass_ as the number of selected spectral features increases, using the SFS optimization procedure and the approach of PROCWT_S3 combined with mPrior_1131 or mPrior_1365. Generally, the improved performance in the estimation of LNC_area_ (NRMSE significantly decreased from 20.94~23.07% to 12.94%~14.49%) and LNC_mass_ (NRMSE dramatically decreased from 32.03~42.21% to 10.11%~10.75%) can be achieved with the use of optimal spectral domains. In particular, the NRMSE values for all cases showed a significant decrease until 5 features, followed by a slower decrease leading to the minimum NRMSE at 28 features (NRMSE = 10.11% to 10.75% for estimating LNC_mass_) or 34 to 45 features (NRMSE = 12.94% to 14.49% for estimating LNC_area_). However, upon inclusion of additional spectral domains related to main water absorption features (such as 1860 to 1899 nm when estimating LNC_area_; 1400 to 1439 nm and 1860 to 1899 nm when estimating LNC_mass_), the NRMSE values increased substantially, particularly for more than 45 features (Fig. 9 and Fig. S2).

## Discussion

### The potential of prior N_struct_ estimation

The N_struct_ parameter in the PROSPECT model [43] is a critical indicator of the internal leaf structure. It reflects the amount of air present in the cell spaces, which dominates the spectral signals across the entire wavelength range (400 to 2500 nm) and contributes to the main uncertainties in the model inversions [29]. Notably, as there is no pigment absorption within the NIR region, the high light scattering within the internal leaf structure leads to the marked impacts of the N_struct_ parameter in this region [25]. This implies that the precise quantification of the N_struct_ parameter is critical to the retrievals of the leaf biochemical constituents in the inversion of PROSPECT. Furthermore, the N_struct_ parameter has been demonstrated to serve as an important indicator of leaf structural diversity [25], nutrient deficit [44], and plant physiology [29].

Qiu et al. [36] and Spafford et al. [25] have proved that the reflectance ratio indices derived from the DHRF spectra at 800 nm (as the optimal waveband for the VNIR region) and 1131 nm (as the optimal waveband for the full-wavelength of 400- to 2500-nm region) are all strongly related to the N_struct_ parameter. This enables prior estimation of N_struct_ using simple reflectance ratio indices. However, these simple reflectance ratio indices may lose their sensitivity to N_struct_, when using leaf BRF spectra affected by leaf specular reflection. With the implementation of the modified ratio indices (i.e., mPrior_800, mPrior_1131 and mPrior_1365) developed in this study (Fig. 6), the improved relationship (*R*^2^ = 0.93 for mPrior_800; *R*^2^ = 0.66 for mPrior_1131; *R*^2^ = 0.74 for mPrior_1365) with N_struct_ potentially allow for the characterization of leaf structure variations and subsequently improve the retrieval performance of constituents. Given the fact that the simulated mPrior_800~N_struct_, mPrior_1131~N_struct_, and mPrior_1365~N_struct_ relationships exhibited no sensitivity to the specular reflectance factor (*b*) (Figs. 6 and 7), as well as the simulated DHRF values equal to the simulated BRF values when *b* is 0, this implies the relationships between the modified indices and N_struct_ can be used to build the unified models across the DHRF and BRF spectra (deducted theoretically in Appendix A).

In this study, spectra simulations were created following the methodology of Féret et al. [2], which encompassed numerous physiological conditions, providing a comprehensive representation of global leaf samples. Additionally, the simulated reflectance spectra were generated with 0.3% random Gaussian noise to mimic the real scenarios. The improved inversion performance of leaf traits by employing modified SIs~N_struct_ relationships was verified in this study, demonstrating that this relationship established with simulated dataset can be applied to the measured dataset. In particular, this dataset includes adequate variations of LNC_area_ (0.065 to 0.404 mg/cm^2^) for *Ginkgo* plants exposed to different N level treatments. In the future, the SIs~N_struct_ relationships based on multiangle BRF data from diverse plant species will require further investigation.

### The selection of the best approach for the constituent retrievals

#### The selection of the prior N_struct_ estimation strategies

In terms of the prior N_struct_ estimation strategies, our results indicate that performing the prior N_struct_ estimation with both standard and modified ratio indices can enhance the accuracy of constituent retrieval, compared to without performing prior N_struct_ estimation. With the use of this No_PriorN_struct_ strategy, the significant underestimation of LNC_area_, LMA and CBC can be found. This bias may be attributed to an overestimation of the N_struct_ parameter, which caused by the additive surface specular reflection effects [31].

In the reflectance simulation, we checked out the simulated samples with extremely lower Cw values (Cw lower than 8 mg/cm^2^) exhibited more dispersed tendency in the relationships between N_struct_ and mPrior_1131, which decreased the fitted *R*^2^_._ This is caused by the fact that much lower Cw values lead to the extremely lower water absorption features around 1925 nm, which give the moderate rise of the reflectance of 1925 nm but the dramatic drop of the mPrior_1131 values. Thus, the N_struct_~mPrior_800 relationships performed better than N_struct_~mPrior_1131 relationships. In particular, the mPrior_1131 index employs 1131 nm, which has been chosen as the optimal band in the calculation of Prior N_struct_ estimation indices within the full visible to shortwave infrared domain (400 to 2500 nm), when combining all datasets in the study of Spafford et al. [25] amounting to 1432 leaf samples, comprising tropical, temperate, and boreal species. This may concentrate the stability of the performance of mPrior_1131 for leaf traits retrieval of *Ginkgo* trees and saplings through different ages, seasons, N fertilizer treatments, and experimental sites. After performing the calculation of the ratio-index formulation, the result indicated that the relationships between N_struct_ and the new proposed index (mPrior_1365) had better *R*^2^ values (*R*^2^ = 0.74) than mPrior_1131 (*R*^2^ = 0.66). This could be explained by the fact that N_struct_~mPrior_1365 relationships can be more robust for the simulated samples with extremely lower Cw values (Cw lower than 8 mg/cm^2^). Specifically, mPrior_1365 provided the best performance in the retrievals of LMA and CBC.

Specifically, the use of modified indices, particularly mPrior_1131 and mPrior_1365, resulted in improved performance compared to standard indices for most constituent traits in our study. This may be attributed to the fact that the modified indices effectively mitigate the specular reflection effects from leaf surface, resulting in a more accurate prior estimation of N_struct_, and thus further enhance the constituent retrieval. In particular, mPrior_1131 and mPrior_1365 utilized 1925 nm (the local minimum reflectance) as the specular reflection factor, effectively suppressing the specular effects in the short-wave infrared region, which is critical for the light absorption of Cw, Cm, and Cp. Furthermore, 1131 nm is recommended as the optimal waveband for the N_struct_ estimation from the full-wavelength of 400- to 2500-nm region [25], which strengthens the stable performance of mPrior_1131 in the model inversion for most constituent trait retrieval. With the use of mPrior_1365, the N_struct_~SIs relationships and also the retrieval of LMA or CBC can be further strengthened. Therefore, we recommend mPrior_1131 or mPrior_1365 to be the default prior N_struct_ estimation strategy.

#### The selection of the optimal approaches

Overall, the performance of standard and modified prior N_struct_ estimation strategies is superior to the strategy without prior N_struct_ estimation (Tables 4 and 5). In particular, the enhanced performance of modified strategies was closely related to the inversion methods utilized. Our findings suggest that modified strategies require inversion methods that consider the specular effects to improve trait retrievals. However, the modified strategies showed no significant improvement in the performance for the STANDARD inversion method (e.g., mPrior_1131 in the study of Spafford et al. [25]), which did not account for the specular effects. This can be mainly explained by the fact that while modified strategies can offer improved N_struct_ estimations, the overestimated N_struct_ values obtained from standard prior N_struct_ estimation strategies tend to offset the adverse impacts of specular reflectance on the trait retrievals when using the STANDARD method (Spafford et al. [25] and Wang et al. [32]). Additionally, using the BRF spectra from the backward scattering direction with leaf clip-equipped spectrometers can minimize the specular effects compared to the forward scattering direction, which may partially explain the lack of significant performance improvement in certain cases when combined with the STANDARD inversion method.

In terms of the STANDARD inversion method (the type of no accounting specular reflection effects), the performance of using modified ratio indices is worse than the standard ratio indices. In particular, when inverting for LMA and CBC, the NRMSE values raised to 47.57% to 62.72% and 43.31% to 59.28%, respectively. In terms of the sPROCOSINE inversion method (the type of specular reflection removal), which requires the estimation of leaf specular reflectance (or specular effects), the performance of the modified strategies is also worse than that of the standard strategy when inverting LMA and CBC. However, the modified strategies can significantly improve the LNC_area_ retrieval success by 26.38% to 44.42%, compared to the standard strategies.

Regarding the inversion methods in the type of specular reflection resistance, most PROCWT-series and PROSDM-series methods combined with the modified strategies generally performed better than those with the standard strategies. In terms of the PROCWT-series methods, combining low-scale PROCWT (PROCWT_S3 and PROCWT_S4) with mPrior_1131 and mPrior_1365 strategies improved the inversion accuracy in LMA (with a reduction in NRMSE by 3.85% to 18.02%), CBC (with a reduction in NRMSE by 6.18% to 17.63%), and LNC_area_ (with a reduction in NRMSE by 10.50% to 16.50%) compared to standard strategies. However, when using mPrior_800 strategy, only achieved improved performance in the LNC_area_ retrieval (NRMSE was reduced by 5.55% to 9.93%). On the other hand, combining moderate-scale (PROCWT_S5) and high-scale (PROCWT_S6) PROCWT with modified strategies did not improve the estimation accuracy in LMA and CBC but only improved estimation accuracy in LNC_area_ (PROCWT_S5 had reduction of 10.20% to 11.22% NRMSE). In terms of PROSDM methods, PROSDM-FED or PROSDM-FMD combining the modified strategies improved the LMA (reduced 2.64% to 14.75% NRMSE) and CBC (reduced 0.50% to 15.49% NRMSE) estimation, whereas both PROSDM-FED and PROSDM-FMD methods failed to achieve better performance in the LNC_area_ estimation. Unlike the specific narrow local maximum absorption features for water and LNC_area_ across the SWIR region that can be well tracked using PROCWT, the absorption features of LMA and CBC are more flat within the range of 1400 to 2000 nm and exhibit a broad rising trend from 2000 to 2500 nm [2]. These absorption features of LMA and CBC can be masked by the strong absorption features of water and thus lead to relatively lower estimation accuracy of LMA and CBC when using PROCWT. In contrast, PROSDM_FMD addresses these issues by considering the accumulative differences rather than the shortest distance between measured and simulated spectra across the entire spectrum. This method reduces BRF-DHRF discrepancies and compensates for first-order derivative limitations, particularly for estimating LMA and CBC with their broad but relatively flat absorption features [31].

After analyzing the performance of 48 approaches (6 strategies × 8 methods), it was determined that combining PROCWT_S3 with mPrior_1365 (mean NRMSE value of 17.23%) or mPrior_1131 (mean NRMSE value of 18.04%) ranked in the top 25%, regardless of in the retrieval of LMA, CBC, and LNC_area_. These 2 approaches are considered as the default optimal approaches. Furthermore, combining mPrior_1131 with PROCWT_S4, PROCWT_S5, PROSDM_FED, PROSDM_FMD, or mPrior_1365 with PROCWT_S5, PROSDM_FED, or PROSDM_FMD can achieve an inversion accuracy that ranks in the top 50%, with mean NRMSE values between 16.53% and 23.87%. Notably, combining mPrior_1365 with PROSDM_FMD achieved the lowest mean NRMSE value of 16.53% among all examined approaches.

### Identification of optimal spectral domains for estimating LNC_area_ and LNC_mass_

With the use of the SFS optimization procedure and the approach of combining PROCWT_S3 with mPrior_1131 or mPrior_1365 for estimating LNC_area_ and LNC_mass_, 28 to 45 optimal spectral features were selected across 5 main isolated spectral domains (Fig. 8). These 5 domains of 1440 to 1539 nm (associated nitrogen absorption features: 1510 nm), 1580 to 1639 nm (associated nitrogen absorption features: 1645 nm), 1900 to 1999 nm (associated nitrogen absorption features: 1980 nm), 2020 to 2099 nm (associated nitrogen absorption features: 2060 nm), and 2120 to 2179 nm (associated nitrogen absorption features: 2130 to 2180 nm), covered the major known nitrogen absorption features primarily attributed to N–H bond stretches within the range of 1400 to 2399 nm [45–47].

In particular, the spectral domain of 2120 to 2179 nm closely align with the strong absorption feature of nitrogen-related protein centered at 2180 nm, as indicated by Curran [45], Fourty et al. [48], Wang et al. [19], and Féret et al. [2]. Additionally, the domain of 2120 to 2179 nm is consistent with the findings of Féret et al. [2], who identified these narrow spectral domains (2100 to 2139 nm and 2160 to 2179 nm) for the protein retrieval based on leaf DHRF spectra. Thus, the domain of 2120 to 2179 nm should be considered in the future estimation of LNC_area_, especially when utilizing BRF spectra obtained from ground-based, unmanned aerial vehicle [UAV]-based, or even airborne or spaceborne sensors. Incorporating this optimal domain can enhance the performance of LNC estimation, which reconfirmed the advantages of optimizing the selection of wavebands using feature selection or waveband weighting methods [2,49]. However, although Féret et al. [2] suggested specific spectral domains (2100 to 2139 nm and 2160 to 2179 nm) for protein or LNC_area_ retrievals, our study found that solely using these recommended wavebands from leaf BRF spectra did not yield optimal performance (*R*^2^ values were negative for most approaches, and thus, the results were not shown). Instead, approaches incorporating the prior N_struct_ estimation generally performed better (*R*^2^ = 0.42 for the best case of standard ratio indices; *R*^2^ = 0.54 for the best case of modified ratio indices) than those without prior N_struct_ estimation (*R*^2^ < 0 for all cases). Specifically, the highest accuracy was achieved by combining the mPrior_1365 strategy with the sPROCOSINE method (*R*^2^ = 0.54, NRMSE = 24.75%).

Regarding the performance for individual spectral features in the SFS procedure (Fig. S2), the LNC estimation based on each spectral feature in the initial model inversion generally performed worse (i.e., with higher NRMSE value in the blue to yellow color) than in subsequent model inversions with more selected spectral subsets. The performance of individual spectral features can be improved by increasing the number of model inversions and consequently including more selected spectral subsets. Moreover, in the SFS procedure, the importance of each spectral feature can vary depending on the number of model inversions performed. In particular, the main water absorption features around 1400 to 1420 nm and 1840 nm were found to result in poor performance, as highlighted by the blue region in Fig. S2. When estimating LNC_mass_, the spectral domain around 2300 nm did not yield satisfactory results, whereas this was not observed in the estimation of LNC_area_. This could be explained by the shadow effects of the main LMA (or CBC) absorption features around 2300 nm [2] on the estimation of LNC_mass_, as this parameter is calculated as the ratio between LNC_area_ and LMA. Our study also found that using intervals of 20 nm (following Féret et al. [2]: NRMSE = 12.94% to 14.49% for estimating LNC_area_; NRMSE = 10.11% to 10.75% for estimating LNC_mass_) for each spectral feature outperformed using intervals of 50 nm (following Wang et al. [32]: NRMSE = 16.64% to 18.12% for estimating LNC_area_; NRMSE = 12.25% to 14.71% for estimating LNC_mass_) (results not shown) but with more computation time (approximately 3 times). The optimal waveband intervals will need to be further investigated in future work.

One needs to pay attention to the relatively lower solar flux energy, and thus low-level signal-to-noise ratio (SNR) for the SWIR region, which includes most of the nitrogen-related (or protein-related) optimal regions. Although these optimal SWIR bands have been proved to be robust in the nitrogen (or protein) estimation based on the leaf-level spectra across various vegetation species and conditions, the weak SNR signals of the SWIR region may lead to uncertainties, especially when performing nitrogen estimation at the canopy level. This can be minimized by using ground-based or UAV-based sensors, which generally have relatively higher SNR signals compared to airborne or spaceborne sensors. Alternatively, although the relationship between chlorophyll and nitrogen may vary among vegetation species, seasons, and conditions, a certain number of previous studies have used chlorophyll content as an indicator of nitrogen content [50], or used as a proxy of photosynthetic nitrogen in the nitrogen allocation model with their counterparts of nonphotosynthetic nitrogen derived from LMA [6]. The rational complementation of chlorophyll monitoring into the nitrogen estimation may prove useful, when using the PROSPECT-PRO model with the separated nitrogen-related protein absorption features [2].

### Limitations and potentials

#### The LNC sample measurements

In this study, only a single nitrogen-to-protein content conversion factor of 4.43 was used, as suggested by Féret et al. [2]. However, this conversion factor may vary across different growth seasons and even other species, as reported by Yeoh and Wee [16], with the range of 3.28 to 5.16. This could be one of the main reasons for the moderately higher uncertainties in the LNC_area_ estimation (Fig. 7) compared to the retrievals of other traits (e.g., LMA and CBC).

Regarding the leaf samples, we collected samples from different aged *Ginkgo* trees (i.e., 14-year-old and 23-year-old) in the *Ginkgo* Forest stands, as well as *Ginkgo* saplings (i.e., 3-year-old and 4-year-old) through different seasons and experimental sites under various nitrogen fertilizer treatments, which hold adequate variations of LNC_area_ (0.065 to 0.404 mg/cm^2^) or LNC_mass_ (7.22 to 34.71 mg/g). The retrieval of LNC_area_ and LNC_mass_ can be optimized by utilizing specific optimal wavebands associated with nitrogen absorption features. This can be achieved using the SFS procedures with mPrior_1131- and mPrior_1365-based N_struct_ prior estimation models derived from spectra simulations. By using spectra simulations that encompass various physiological conditions and include 0.3% random Gaussian noise, this approach can mimic real scenarios and potentially yield robust performance for different plant species. Indeed, the difference in the growth seasons and tree species can lead to larger variation in LNC_area_ and LNC_mass_. In this study, samples collected in July exhibited higher average LNC_mass_ values (23.83 mg/g) compared to those collected from August to September, where average LNC_mass_ values ranged from 18.97 to 21.06 mg/g. Younger leaves generally have higher LNC_mass_ values, which tend to decrease as the leaves mature due to nitrogen dilution effects resulting from the accumulation of leaf biomass [51]. To further address this issue, future studies will include more samples from different tree species and even other vegetation types, covering a wider range of growth seasons and leaf ages, particularly during the early rapid growth stage.

#### The leaf BRF measurements

As compared to the forward scattering direction, leaf surfaces exhibit less significant anisotropy and BRF characteristics in the backward scattering direction [11,40,52–54]. Indeed, when employing proximal remote sensing techniques, such as spectrometers equipped with leaf clips or close-range imaging spectrometers [37,39], the surface of leaves can be perceived as uneven or undulating at a small scale (finer than 1 mm), due to the presence of waxy cuticles and epidermal roughness. This uneven surface comprises tilted mirror-like tiny facets [40], as depicted by the “V”-shape lines in Fig. 3C in the study of Zhou et al. [33]. These facets enable specular reflection of photons in the backward scattering direction, although with less BRF characteristics compared to the forward scattering direction. Younger leaves typically exhibit smoother surfaces, leading to more pronounced specular reflections during leaf reflectance measurements. In contrast, older leaves tend to have rougher surfaces, which may diminish the intensity of specular reflections. Despite the above age-related variations in fluctuation levels of leaf surface wax layer [33], the impact of specular reflection on remote sensing measurements is also influenced by the specific measurement techniques and angles employed [11]. Further studies should examine the extent to which spectral characteristics vary with leaf age, particularly concerning specular reflections. Furthermore, Fig. 4 in the study of Wan et al. [31] illustrates that the contribution of the specular reflection factor to the BRF spectra can reach 20% to 100% in the low-reflectance bands at the SWIR regions (1400 to 2400 nm), especially for the range of 1900 to 2400 nm where it can exceed 40%. This suggests that the contribution of specular reflection to the BRF spectra could be significant around 1900 to 2400 nm, which includes the most sensitive regions (2100 to 2300 nm) for retrieving protein-related LNC with the PROSPECT-PRO model [2]. Therefore, the presence of specular reflection in the BRF spectra, originating from the backward scattering direction in this study, is substantial enough to noticeably impact the leaf spectra [39]. Compared to leaf clip-equipped spectrometers with fixed illumination–observation geometry, multiangle spectral BRF data is more useful for practical remote sensing applications and hyperspectral imaging mapping [11,54,55]. However, the variability in illumination–observation geometry makes the BRF signals complex, limiting the ability to directly invert the PROSPECT-PRO model from multiangle BRF spectra, especially the difference between the simulated specular components in the DHRF and multiangle BRF spectra cannot be simply expressed with a constant of specular factor *b*. Instead, it is necessary to carefully extract leaf traits from the multiangle BRF spectra by combining the PROSPECT-PRO model with the bidirectional reflectance distribution function model [40], which expresses the directional component by accounting for the illumination and observation geometries, as well as the wax refractive index and surface roughness parameter. These combined models could be somewhat overly parameterized to different extents and thus lead to ill-posed inversion issues with worse estimation performance [37]. These issues need to be fully addressed in the future work with more efficient inversion methods.

#### The potential of using modified prior N_struct_ estimation strategies

Figure 3 reveals that LNC_mass_ and LNC_area_ increased with higher nitrogen fertilizer levels, reaching a peak at the N3 level (675 kg/hm^2^). This positive response emphasizes the critical role of nitrogen in supporting the growth and development of *Ginkgo* saplings. However, the subsequent stabilization or slight decrease in LNC values from N3 (675 kg/hm^2^) to N5 (1125 kg/hm^2^) suggests that excessive nitrogen may not provide additional benefits and could potentially harm plant health through nitrogen saturation or leaching [4,5]. In this context, remote sensing offers a promising solution to address the limitations of traditional nitrogen management practices, which often rely on visual diagnosis or delayed laboratory analysis. As highlighted in our study, the use of spectral techniques for LNC estimation provides a rapid, nondestructive approach that can potentially improve the precision of nitrogen fertilizer applications in *Ginkgo*, especially when combined with mechanistic modeling approaches. This would allow *Ginkgo* growers to effectively monitor nitrogen levels throughout critical growth stages, ranging from the early bud development stage to the plant maturity stage, thereby avoiding overfertilization or nitrogen deficiency, both of which can negatively impact tree health and biomass accumulation. Moreover, given that nitrogen is closely tied to the synthesis of medicinal compounds in *Ginkgo* leaves [56], optimizing nitrogen application through precise monitoring can also help ensure the production of valuable compounds, such as flavonoids [33,57]. This highlights the utility of integrating remote sensing tools for more targeted and efficient nutrient management in *Ginkgo*, ultimately reducing both environmental impact and resource wastage.

The strategy of Prior N_struct_ estimation proposed in our study is relatively straightforward to apply based on commonly used BRF spectra, with no marked loss in computing efficiency and even more efficient than the strategy without the Prior N_struct_ estimation, which requires inverting N_struct_ together with other traits. In comparison to the DHRF spectra obtained by integrating sphere equipped spectrometers, BRF spectra are easier and more practical to collect for common scenarios, such as using leaf clip-equipped spectrometers [30,33], close-range hyperspectral imaging systems [51,58], and even near-ground UAV imaging systems [57,59]. Therefore, more efforts should be paid to the use of BRF spectra in the Prior N_struct_ estimation and the further retrieval of constituents (e.g., LNC). Furthermore, the different performance in the LNC estimation using the novel ratio indices between DHRF spectra and BRF spectra also needs to be examined in our future work.

The novel ratio indices developed in this study were built based on 800 and 1131 nm, which are highly related to the N_struct_ variation reported in previous researches [25,36], while avoiding high water absorption regions [60–62]. These characteristics may potentially help improve the N_struct_ estimation in leaf- and canopy-level applications. At the leaf level, different cell types (e.g., surface epidermal cells and palisade columnar or spongy spherical mesophyll cells), are currently assumed to be identical, despite their anatomical differences that affect light path length [25,30,36,63]. Thus, improvements to N_struct_ parameter characterization are needed to represent cell-specific structural variation within leaves in PROSPECT, to further enhance the estimation of constituents [36]. At the canopy level, the confounding effects of canopy structure and soil background may lead to uncertainties in the N_struct_ estimation and result in a bias in the further constituent retrievals. To effectively scale up our findings for practical use in forest, it is crucial to explore the potential of implementing the inversion of PROSPECT-PRO coupled with canopy RTMs, such as the 4SAIL model, to estimate LNC based on canopy-level BRF spectra. While our spectral data were collected in a controlled lab environment, the methodologies developed can be adapted for field measurements using close-range or airborne spectrometers. With the use of airborne hyperspectral imagery, Berger et al. [18] highlighted the great potential of performing PROSAIL-PRO (i.e., the PROSPECT-PRO model coupled with the SAIL model) inversion to estimate nitrogen content in crop canopies (i.e., the product of LNC and LA index), suggesting that similar methodologies could be adapted for assessing nitrogen levels in forests. They confirmed the main optimal bands were located at the SWIR region, with the most important bands of 2124 and 2234 nm related to nitrogen (or protein) absorption features, except for one NIR band of 783 nm related to the canopy structure. Alternatively, to alleviate the structure effects, the signals of LNC could be enhanced by using the strategy of canopy scattering coefficient [64,65] based on the canopy-level observation data. By integrating these methods into field studies, we can effectively bridge the gap between laboratory findings and practical applications, leading to improved nitrogen management strategies in forests.

## Conclusion

This study proposed an advanced BRF spectra-based approach for the prior estimation of the N_struct_ structure parameter to improve the retrieval of LNC in *Ginkgo* trees. Three modified ratio indices (i.e., mPrior_800, mPrior_1131, and mPrior_1365) and their corresponding linear models with N_struct_, were developed and evaluated for their effectiveness in the LNC retrieval, through the inversion of the PROSPECT-PRO model. The proposed strategy of prior N_struct_ estimation using modified ratio indices showed the best performance in constituent estimation compared to using standard ratio indices or not performing prior N_struct_ estimation. By using the SWIR band of 1131 or 1365 nm, mPrior_1131 or mPrior_1365 out of the prior N_struct_ estimation strategies yielded reliable performance for all constituents examined in this study. The use of PROSDM_FMD combined with mPrior_1365 provided the best performance for retrieving LMA and CBC, while the PROCWT_S3 method combined with mPrior_800 outperformed other approaches in the retrieval of LNC_area_. Our results also revealed that the optimal estimation of LNC_area_ and LNC_mass_ shared 5 main common domains of 1440 to 1539 nm, 1580 to 1639 nm, 1900 to 1999 nm, 2020 to 2099 nm, and 2120 to 2179 nm. These findings highlighted the potential of using the proposed BRF spectra-based approach for the prior estimation of leaf structure to improve constituent retrieval, especially for the LNC estimation. Although this study focused on *Ginkgo* leaves, the findings may be applicable to other vegetation types based on the inversion of the PROSPECT-PRO model. Moreover, this study provides new insights into the impact of N_struct_ prior estimation on the LNC retrieval.

## Data Availability

The datasets analyzed during the current study are available from the corresponding author upon reasonable request.

## Appendix A. The deducing process of developing modified ratio indices for the prior N_struct_ estimation

According to the hypothesis proposed by Spafford et al. [25], N_struct_ can be linked to the ratio indices at the minimum absorption domains, in which the absorption can be negligible in the minimum absorption domains. The equation is listed as below:

$$N_{\text{struct}}\propto \frac{R_{\text{minA}}}{T_{\text{minA}}}\approx \frac{R_{\text{minA}}}{1-R_{\text{minA}}}\approx \frac{1-T_{\text{minA}}}{T_{\text{minA}}}$$ (A1)

where $R_{\text{minA}}$ and $T_{\text{minA}}$ represent the reflectance and transmittance at the waveband with minimum absorption, respectively.

When employing the reflectance of 800 nm (the optimal minimum absorption band for the 400-800 nm region) and 1131 nm (the optimal minimum absorption band for the 400-2500 nm region) into the ratio-index related to N_struct_, the Eq. A1 can be revised as below:

$$N_{\text{struct}}\propto \frac{R_{\text{minA}}}{T_{\text{minA}}}\approx \frac{R_{\text{minA}}}{1-R_{\text{minA}}}=\frac{{\text{DHRF}}_{800}}{1-{\text{DHRF}}_{800}}$$ (A2)

$$N_{\text{struct}}\propto \frac{R_{\text{minA}}}{T_{\text{minA}}}\approx \frac{R_{\text{minA}}}{1-R_{\text{minA}}}=\frac{{\text{DHRF}}_{1131}}{1-{\text{DHRF}}_{1131}}$$ (A3)

However, compared to DHRF spectra, the BRF spectra are more sensitive to the specular reflection. The BRF spectra can be connected to the DHRF spectra [30,32,37] with the simulated relationships as below:

$${\mathrm{BRF}}_{\lambda }=\left(\frac{\text{cos}\theta_{i}}{\text{cos}\theta_{s}}\right)\;\left[{\text{DHRF}}_{\lambda }+b\right]$$ (A4)

where $\theta_{i}$ and $\theta_{s}$ are the light incident angle (i.e., angle between the leaf normal and the light source), and the illumination zenith angle, respectively. *b* represents a wavelength-independent constant of specular reflection factor, which corresponds to the difference in the specular reflectance components between leaf BRF and DHRF spectra [30,32,37,40].

Given the fact that the light incident angle $\theta_{i}$ and the illumination zenith angle $\theta_{s}$ were set to the same value of 12*°* in the acquisition geometry of leaf clip (with the relative azimuth angle of 0*°*), Eq. (A4) can be simplified as below:

$${\text{DHRF}}_{\lambda }={\mathrm{BRF}}_{\lambda }-b$$ (A5)

where λ represents the wavelength and b represents a constant of specular reflection factor.

According to Eq. (A5), Eqs. (A2-A3) can be rewritten as below:

$$N_{\text{struct}}\propto \frac{{\text{DHRF}}_{800}}{1-{\text{DHRF}}_{800}}=\frac{{\text{BRF}}_{800}-b}{1-\left({\text{BRF}}_{800}-b\right)}$$ (A6)

$$N_{\text{struct}}\propto \frac{{\text{DHRF}}_{1131}}{1-{\text{DHRF}}_{1131}}=\frac{{\text{BRF}}_{1131}-b}{1-\left({\text{BRF}}_{1131}-b\right)}$$ (A7)

As followed the strategy in Sims and Gamon [41] and Li et al. [30], to assume the reflectance of 445 nm (maximum absorption within VNIR region) and 1925 nm (maximum absorption within SWIR region) as the constant of specular reflectance factor *b* in the VNIR region (400-800 nm) and the SWIR region (1000-2500 nm), respectively. The Eqs. (A6-A7) can be revised as below:

$$\begin{array}{c} N_{\text{struct}}\propto \frac{{\text{DHRF}}_{800}}{1-{\text{DHRF}}_{800}}\\ =\frac{{\text{BRF}}_{800}-b}{1-\left({\text{BRF}}_{800}-b\right)}\approx \frac{{\text{BRF}}_{800}-{\text{BRF}}_{445}}{1-\left({\text{BRF}}_{800}-{\text{BRF}}_{445}\right)} \end{array}$$ (A8)

$$\begin{array}{c} N_{\text{struct}}\propto \frac{{\text{DHRF}}_{1131}}{1-{\text{DHRF}}_{1131}}\\ =\frac{{\text{BRF}}_{1131}-b}{1-\left({\text{BRF}}_{1131}-b\right)}\approx \frac{{\text{BRF}}_{1131}-{\text{BRF}}_{1925}}{1-\left({\text{BRF}}_{1131}-{\text{BRF}}_{1925}\right)} \end{array}$$ (A9)

According to Eq. (A5), the difference in the DHRF spectra between two wavelengths equals to the difference in the BRF spectra. This can be listed as below:

$$\begin{array}{c} {\text{DHRF}}_{800}-{\text{DHRF}}_{445}=\left({\mathrm{BRF}}_{800}-b\right)-\left({\mathrm{BRF}}_{445}-b\right)\\ ={\mathrm{BRF}}_{800}-{\mathrm{BRF}}_{445} \end{array}$$ (A10)

$$\begin{array}{c} {\text{DHRF}}_{1131}-{\text{DHRF}}_{1925}=\left({\mathrm{BRF}}_{1131}-b\right)-\left({\mathrm{BRF}}_{1925}-b\right)\\ ={\mathrm{BRF}}_{1131}-{\mathrm{BRF}}_{1925} \end{array}$$ (A11)

According to Eqs. (A10-A11), Eqs. (A8-A9) can be rewritten as below:

$$\begin{array}{c} N_{\text{struct}}\propto \frac{{\text{BRF}}_{800}-{\text{BRF}}_{445}}{1-\left({\text{BRF}}_{800}-{\text{BRF}}_{445}\right)}\\ =\frac{{\text{DHRF}}_{800}-{\text{DHRF}}_{445}}{1-\left({\text{DHRF}}_{800}-{\text{DHRF}}_{445}\right)} \end{array}$$ (A12)

$$\begin{array}{c} N_{\text{struct}}\propto \frac{{\text{BRF}}_{1131}-{\text{BRF}}_{1925}}{1-\left({\text{BRF}}_{1131}-{\text{BRF}}_{1925}\right)}\\ =\frac{{\text{DHRF}}_{1131}-{\text{DHRF}}_{1925}}{1-\left({\text{DHRF}}_{1131}-{\text{DHRF}}_{1925}\right)} \end{array}$$ (A13)

Thus, the relationships between the modified indices and N_struct_ can be used to build the unified models across the DHRF and BRF spectra theoretically.
