# Supplementary material for: Coupling PROSPECT with Prior Estimation of Leaf Structure to Improve the Retrieval of Leaf Nitrogen Content in Ginkgo from Bidirectional Reflectance Factor Spectra
Source: Plant Phenomics. 2024 Dec 13;6:0282. doi: 10.34133/plantphenomics.0282 (PMC11641793; doi:10.34133/plantphenomics.0282)
Supplement: Supplementary 1 — Figs. S1 and S2 [file plantphenomics.0282.f1.docx]

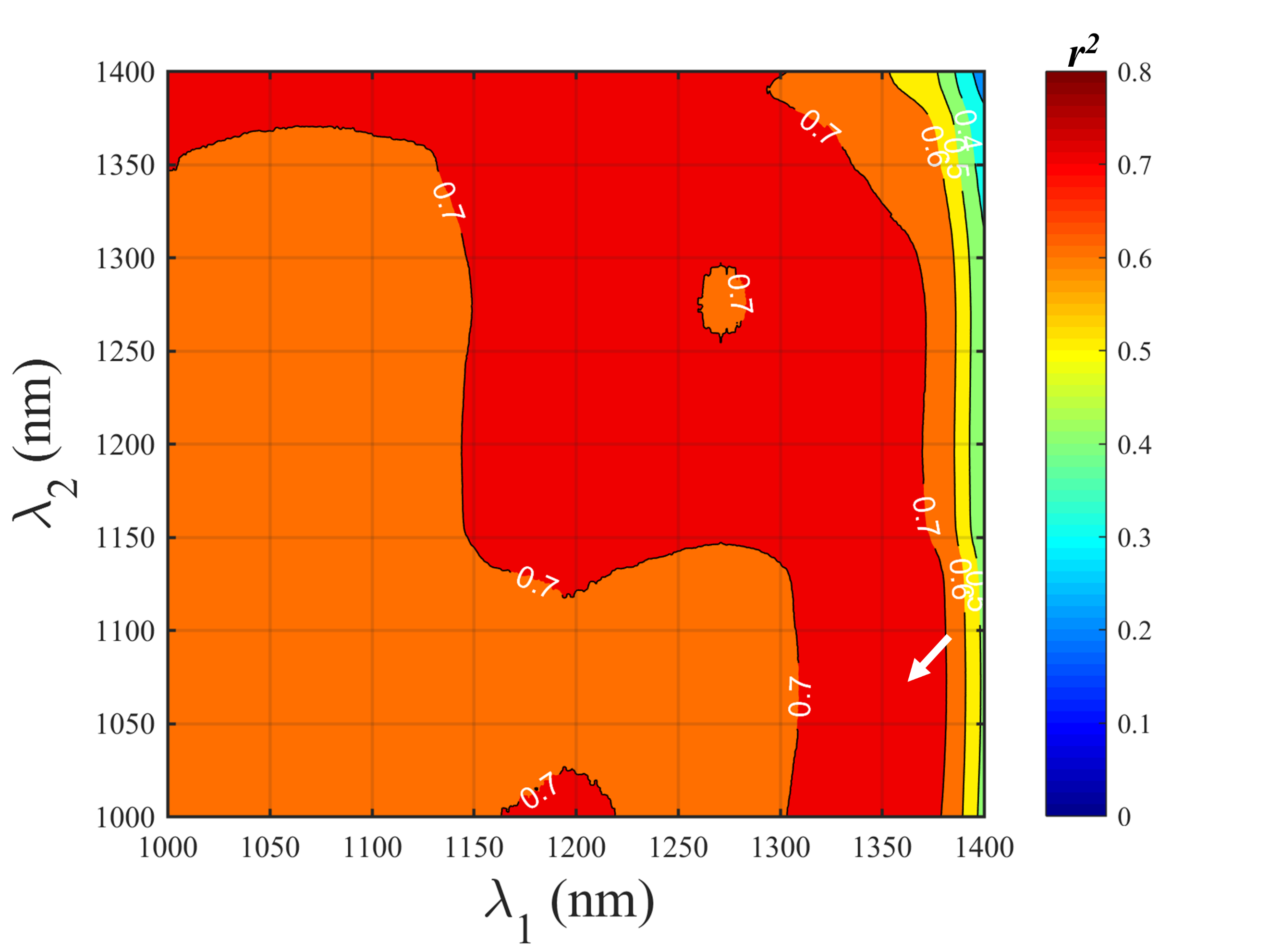


**Fig. S1** The contour maps of square Pearson correlation coefficients (*r^2^*) between Ns and the modified ratio-indices in the SWIR sub-regions from 1000-1400 nm. The white arrow in the map represents the highlight spot in the relationships between Ns and the modified ratio-indices.
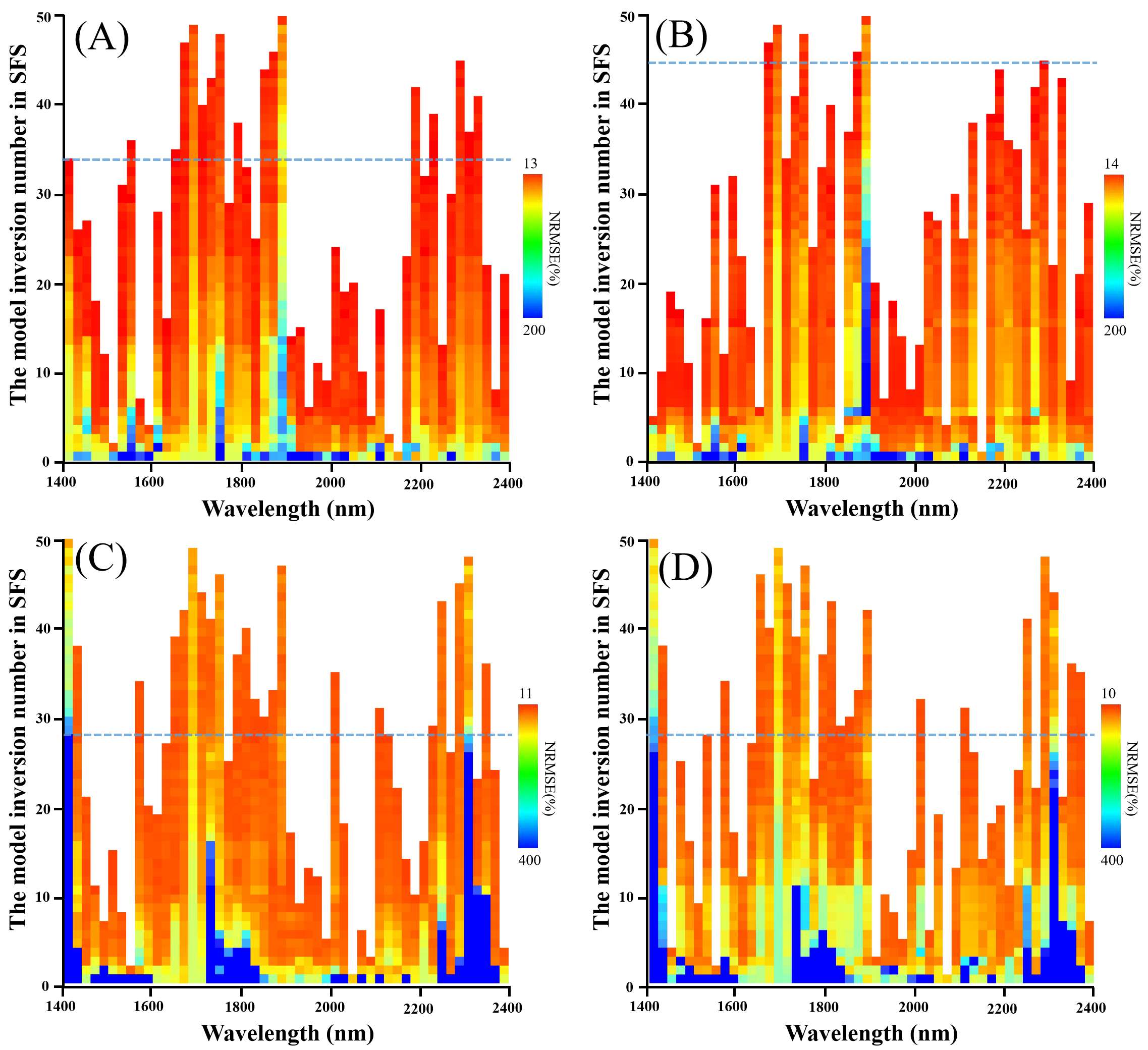


**Fig. S2.** The NRMSE values for estimating LNC_area_ and LNC_mass_ in the selection of optimal spectral domains using the technique of sequential forward feature selection (SFS), as well as the approach of combining PROCWT_S3 with mPrior_1131 (A; C) or mPrior_1365 (B; D). The X-axis represents the wavelength added to the selected spectral domains in the SFS procedure. The Y-axis represents the model inversion number in the SFS procedure. The blue dotted lines in each subfigure represent the number of model inversions which lead to the minimum NRMSE among all runs. The maximum inversion number value (i.e., the maximum bar value) for each spectral subset represents the wavelength added to the selected domains, which lead to minimum NRMSE in each run (corresponding to individual model inversion numbers). The spectral subsets those hold the maximum bar value not exceeding the blue dotted lines represent the selected optimal spectral domains.
